# FTSJ3‐Mediated 2′‐O‐Methylation of HSPA5 mRNA Stabilizes BOP1 via SUMOylation to Drive DRP1‐Dependent Mitochondrial Fission and Fatty Acid Oxidation in Lung Squamous Cell Carcinoma

**DOI:** 10.1002/advs.77666

**Published:** 2026-09-08

**Authors:** Qin Hu, Jiaheng Lin, Yifei Zhu, Ying Liu, Guoren Zhou

**Affiliations:** ^1^ Department of Oncology The Affiliated Cancer Hospital of Nanjing Medical University Jiangsu Cancer Hospital Jiangsu Institute of Cancer Research Nanjing Jiangsu People's Republic of China; ^2^ The Fourth Clinical Medicine College Nanjing Medical University Nanjing Jiangsu People's Republic of China; ^3^ Center for Global Health The Key Laboratory of Modern Toxicology Ministry of Education School of Public Health Suzhou Institute for Advanced Study of Public Health Gusu School Nanjing Medical University Nanjing Jiangsu People's Republic of China; ^4^ Department of Oncology Jiangsu Cancer Hospital The Affiliated Cancer Hospital of Nanjing Medical University Jiangsu Institute of Cancer Research Nanjing Jiangsu People's Republic of China

**Keywords:** 2′‐O‐methylation, BOP1, fatty acid oxidation, FTSJ3, liposome delivery, lung squamous cell carcinoma, SUMOylation

## Abstract

Lung squamous cell carcinoma (LUSC) is a highly aggressive malignancy with a dismal prognosis and limited targeted therapies. While post‐translational and RNA modifications are implicated in tumor progression, their precise roles in LUSC remain elusive. Here, we identify the RNA methyltransferase FTSJ3 as a critical oncogenic driver. FTSJ3 is markedly upregulated in LUSC tissues, and its high expression correlates with poor patient survival. Mechanistically, FTSJ3 catalyzes 2′‐O‐methylation of HSPA5 mRNA, leading to transcript stabilization and increased protein abundance of the molecular chaperone HSPA5. Elevated HSPA5 facilitates the SUMOylation and subsequent stabilization of the ribosomal biogenesis factor BOP1. This modification event activates the DRP1/SREBP1 signaling axis, driving excessive mitochondrial fission and reprogramming cellular metabolism towards fatty acid oxidation (FAO), providing essential energy and biomolecular precursors for tumor growth. Silencing of FTSJ3 disrupts this HSPA5/BOP1/DRP1 cascade, inhibiting mitochondrial fragmentation, FAO, and the proliferation, migration, and invasion of LUSC cells. In vivo, delivery of FTSJ3‐specific siRNA via liposomal nanoparticles suppresses tumor growth and metastasis in LUSC models. Our findings delineate a novel FTSJ3‐HSPA5‐BOP1‐DRP1‐SREBP1 axis that integrates RNA methylation with SUMOylation, mitochondrial dynamics and lipid metabolism to promote LUSC progression, nominating FTSJ3 as a therapeutic target for this cancer.

## Graphical abstract

The RNA methyltransferase FTSJ3 drives lung squamous cell carcinoma progression by catalyzing 2′‐O‐methylation of HSPA5 mRNA, which stabilizes BOP1 through SUMOylation and activates DRP1/SREBP1‐mediated mitochondrial fission and fatty acid oxidation. Targeting this axis with liposomal siRNA potently suppresses tumor growth and metastasis

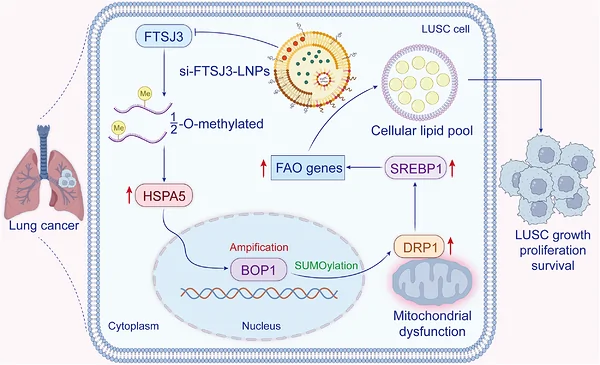

## 1 Introduction

Lung cancer remains the leading cause of cancer‐related death worldwide, with an estimated 1.8 million fatalities annually [1]. Non‐small‐cell lung cancer (NSCLC) accounts for >85% of cases, and lung squamous cell carcinoma (LUSC) represents 30–40% of NSCLC, making it the second most common histological subtype [2]. LUSC typically presents at an advanced stage and is characterized by high genomic heterogeneity, extensive DNA damage, and a heavy smoking‐associated mutational burden [3]. These features have hampered the identification of actionable driver mutations, leaving LUSC with fewer approved targeted agents than lung adenocarcinoma. Consequently, the 5‐year overall survival (OS) rate remains <18%, and virtually all patients eventually relapse with therapy‐resistant disease [4, 5]. There is therefore an urgent unmet need to elucidate the molecular underpinnings that sustain LUSC progression and to identify tractable therapeutic vulnerabilities.

Post‐transcriptional and epigenetic modifications have emerged as central regulators of malignant phenotypes. Among these, RNA chemical modifications—collectively termed the “epitranscriptome”—control RNA fate decisions, including splicing, stability, translation, and protein–RNA interactions [6]. Over 170 distinct RNA modifications have been catalogued; of them, N6‐methyladenosine (m6A), 5‐methylcytidine (m5C), and 2′‐O‐methylation are the most frequently dysregulated in cancer [7]. In LUSC, altered m6A writer/reader proteins (e.g., FTO, YTHDF2) promote proliferation and immune evasion [8, 9], whereas m5C regulators such as ALYREF correlate with poor prognosis [9]. Despite these advances, the biological significance of RNA ribose 2′‐O‐methyltransferases—particularly the human orthologue of yeast FTSJ3—remains virtually unexplored in LUSC. FTSJ3 was originally identified as an HIV TAR RNA‐binding partner [10] and was recently shown to facilitate immune evasion in malignant tumors via methylation‐dependent dampening of type‐I interferon signaling [11]; however, its potential oncogenic role in LUSC remains a complete enigma. Therefore, this study aims to investigate the correlation and potential mechanisms of methylation modification‐related genes (MRGs) in LUSC prognosis using bioinformatics databases.

Small‐ubiquitin‐like modifier (SUMO) conjugation is a reversible post‐translational modification (PTM) that modulates protein stability, sub‐cellular localization, and protein–protein interactions without targeting substrates for degradation [12]. Global SUMOylation is frequently upregulated in lung cancer cell lines, and lncRNA SUMO1P3 is an independent adverse prognostic factor in lung adenocarcinoma [13]. In LUSC, only SENP2‐mediated de‐SUMOylation has been linked to aberrant squamous differentiation [14], while the landscape of SUMOylation (the SUMOylome) and its functional consequences in LUSC are vastly underexplored, representing a missed opportunity for therapeutic discovery. Importantly, crosstalk between RNA methylation and SUMOylation has been reported in gastric cancer, where SUMOylated NSUN2 enhances m5C deposition and tumor growth [15, 16, 17, 18], implying that similar regulatory circuits may operate in LUSC.

Mitochondrial dynamics—controlled by balanced fusion and fission events—govern bioenergetic efficiency, redox homeostasis, and apoptotic priming. Excessive mitochondrial fission, driven by the GTPase DRP1, triggers fragmentation, metabolic re‐wiring, and therapy resistance in multiple cancers [19, 20, 21, 22, 23]. These findings suggest that mitochondrial dysfunction plays a promotive role in NSCLC progression, but research on mitochondrial fission in LUSC is limited. In NSCLC, DRP1‐mediated fragmentation fosters immune evasion and limits ROS‐induced ferroptosis [24, 25]. Concomitantly, tumor cells upregulate fatty‐acid oxidation (FAO) to sustain ATP production and redox balance under nutrient stress [26, 27, 28]. Although FAO has been implicated in lung adenocarcinoma stemness [62], its regulatory circuitry in LUSC remains undefined. Critically, whether RNA‐modifying enzymes couple mitochondrial fission to FAO activation in LUSC is entirely unexplored.

We therefore posit that FTSJ3 is a central orchestrator of a novel oncogenic axis in LUSC. Our model proposes a linear cascade: FTSJ3‐catalyzed 2′‐O‐methylation of HSPA5 mRNA enhances transcript stability, thereby elevating HSPA5 protein abundance, which in turn facilitates SUMOylation‐dependent stabilization of the ribosomal factor BOP1. Stabilized BOP1 subsequently upregulates DRP1 expression, thereby promoting mitochondrial fission and SREBP1‐mediated fatty‐acid oxidation (FAO). This metabolic reprogramming is essential for tumor progression (Figure 7Q).

The present study integrates multi‐omics datasets, functional genomics, and therapeutic RNA‐delivery platforms to (i) characterize FTSJ3 expression and prognostic significance in LUSC; (ii) delineate the molecular axis linking FTSJ3‐mediated RNA methylation to SUMOylation, mitochondrial fragmentation, and FAO; and (iii) evaluate the translational potential of targeting this cascade with siRNA‐loaded liposomes. Given the paucity of targeted options and the inevitability of resistance in LUSC, the identification of the FTSJ3‐driven axis and its vulnerability to liposomal siRNA therapy directly addresses this clinical imperative. Our approach not only targets a novel oncogenic driver but also utilizes a delivery platform capable of overcoming biological barriers, thereby translating a fundamental mechanistic insight into a tangible therapeutic candidate. Our findings uncover a therapeutically actionable epigenetic‐metabolic circuitry that may improve clinical outcomes for LUSC patients.

## 2 Materials and Methods

### 2.1 Data Source

The GSE30219 dataset was downloaded from the GEO database (https://www.ncbi.nlm.nih.gov/geo/), which includes 61 LUSC tissue samples and 14 adjacent non‐cancerous tissue samples (GPL570) [29]. Due to the lack of prognostic information for LUSC in other databases, the 61 LUSC tissue samples from the GSE30219 dataset were randomly divided into two groups in a 7:3 ratio. 70% of the samples were designated as the training set for differential gene identification and risk model construction, while 30% of the samples were used as the validation set for risk model validation. The study focused on 10 types of RNA modification‐related genes (RNA‐MRGs), comprising 150 non‐redundant genes in total [30].

### 2.2 Gene Screening

1. Identification of differentially expressed genes (DEGs): To identify differentially expressed genes between the LUSC group and the control group, the “limma” package was utilized to compare expression differences in the GSE30219 dataset (disease group vs. control group). The selection criteria were set as |log2 FoldChange| ≥ 0.5 and p < 0.05. Volcano plots and heatmaps were generated using the R packages “ggplot2” and “ComplexHeatmap,” respectively, based on the results.
2. Identification of Candidate Genes: To obtain genes associated with both LUSC and the 10 types of RNA‐MRGs, a Venn diagram was created using the “ggVenndiagram” package. The intersection of 4,576 DEGs from the GSE30219 dataset and 150 RNA‐MRGs was identified, and these intersecting genes were designated as candidate genes.
3. GO/KEGG Enrichment Analysis and PPI Network Construction: The candidate genes were subjected to GO and KEGG enrichment analyses using the R package “clusterProfiler” to investigate the biological functions involved. The selection criterion for KEGG enrichment analysis was set at p value < 0.05. A protein‐protein interaction (PPI) network for the candidate genes was constructed using the STRING database (https://string‐db.org), with a confidence score of 0.4, resulting in a network comprising 31 nodes and 178 edges.

### 2.3 Construction and Validation of the Prognostic Risk Model

The GSE30219 dataset, comprising 61 LUSC patients, was randomly divided into two parts in a 7:3 ratio, designated as the “training set (N = 43)” and the “validation set (N = 18).” This division was used to construct and validate a prognostic model for predicting the survival of LUSC patients. Based on the previously identified candidate genes, univariate Cox regression analysis was performed using the “survival” package, with a significance threshold of p < 0.2. The proportional hazards (PH) assumption test was conducted on the results of the univariate regression analysis (P > 0.05). Genes that passed the PH assumption test were considered to have prognostic value. Further gene selection was performed using the “glmnet” package to conduct Lasso regression analysis on the genes that passed the PH assumption test.

To explore the correlation between four prognostic genes (NCBP2, DNMT3B, FTSJ3, and IGF2BP2) and differential immune cells, Spearman correlation analysis was conducted using the R package “psych” on LUSC samples with survival information from the high‐ and low‐risk groups in the GSE30219 training set. The correlation coefficients were obtained with a threshold of |Cor| > 0.3 and p < 0.05.

The infiltration abundance of immune cells was visualized using stacked bar plots generated with the R package “ggplot2,” illustrating the immune infiltration abundance in high‐ and low‐risk groups.

### 2.4 Analysis of Single‐Cell RNA Sequencing Data

We integrated single‐cell RNA sequencing (scRNA‐seq) data from five lung squamous cell carcinoma (LUSC) specimens (GSE127465). Seurat package (v4.0) was used for data integration and quality control analysis. Cell quality was evaluated by calculating the percentages of mitochondrial genes (^ Mt ‐), ribosomal genes (^ Rp [sl]), and hemoglobin genes (^ Hb [^ (p)]). The quality control standards were set as follows: gene count<3000, UMI count<50000, mitochondrial gene proportion<20%, ribosomal gene proportion>3%, and hemoglobin gene proportion<1%. LogNormalize method (scale factor = 10000) was used to normalize gene expression. The first 2000 highly variable genes were identified for downstream analysis and all genes were standardized. Principal component analysis (PCA) was used to determine the optimal principal component score based on cumulative contribution rate and variation between principal components. The Harmony algorithm was used to correct batch effects between samples, with “orig.ident” as the grouping variable. A K‐nearest neighbor graph was constructed based on corrected principal components, and the Louvain algorithm was used for cell clustering, testing resolution parameters within the range of 0.01–1.0. The scDblFinder package was used to identify and remove doublet cells (dbr = 0.08), while retaining single cells for subsequent analysis.

### 2.5 qRT‐PCR Detection

Total RNA was extracted from tumor tissue and adjacent tissue from patients with LUSC using TRIzol reagent. RNA concentration was assessed by fluorescence. Reverse transcription was performed using Sure Script‐First‐strand‐cDNA‐synthesis‐kit from Wuhan Xavier, according to the manufacturer's protocol. Briefly, kit components were thawed to room temperature, centrifuged briefly, and kept on ice prior to use in the reverse transcription reaction. Quantitative PCR (qPCR) was performed using the following reaction mixture: 3 µL of cDNA template, 5 µL of 2× SYBR Green Master Mix, 1 µL each of forward and reverse primers (10 µM). Primer sequences used in this study are provided in Table S1.

### 2.6 Reagents

Radioimmunoprecipitation assay (RIPA) lysis buffer, TRIzol reagent, and the BCA protein assay kit were purchased from Yamei Biotechnology (Shanghai, China). DMSO and H&E staining reagents were supplied by Aibo Kang (Shanghai, China). Primary antibodies were obtained from the indicated suppliers: FTSJ3 (for WB and IHC, 50607S, Cell Signaling Technology; for IF, ab186009, Abcam), BOP1 (for WB, ab86653, Abcam; for IHC and IF, sc‐28383, Santa Cruz Biotechnology), SUMO‐2/3/4 (sc‐393144, Santa Cruz Biotechnology), DRP1 (ab184247, Abcam), SREBP1 (sc‐366, Santa Cruz Biotechnology), HSPA5 (for WB, ab32618, Abcam; for IF, sc‐10586, Santa Cruz Biotechnology), FASN (ab207369, Abcam), ACC1 (A300‐986A, Thermo Fisher Scientific), ACLY (67166‐1‐Ig, Proteintech), SCD (sc‐515875, Santa Cruz Biotechnology), CPT1A (ab128568, Abcam), and GAPDH (ab157156, Abcam). For WB, HRP‐conjugated goat anti‐rabbit IgG (31460, Invitrogen) was used as the secondary antibody. For IF, Alexa Fluor Plus 488–conjugated goat anti‐rabbit IgG (A32731, Thermo Fisher Scientific), Alexa Fluor Plus 594–conjugated goat anti‐mouse IgG (A32742, Thermo Fisher Scientific), and Alexa Fluor Plus 647–conjugated donkey anti‐goat IgG (A21447, Thermo Fisher Scientific) were employed. DAPI‐containing fluorescence mounting medium was obtained from Abcam (ab104139).

### 2.7 Mice

Male Balb/c and C57BL/6 mice (6–8 weeks old, weight 22–25 g) were purchased from Beijing Vital River Laboratory Animal Technology Co., Ltd. Mice were housed in an SPF‐level laboratory animal room at 23 ± 3°C, with free access to food and water.

All experimental procedures complied with the guidelines of Nanjing Medical University Institutional Animal Care and Use Committee, and the study strictly adhered to approved animal experimentation protocols (Animal Ethics Approval Number: IACUC‐2509022).

### 2.8 Cell Culture

The LUSC cells H520 and SKMES were cultured with RPMI‐1640 medium supplemented with 10% fetal bovine serum (Hyclone, Logan, UT, USA) and penicillin/streptomycin (100 mg/mL) at 37°C and 5% CO_2_. Both cell lines were mycoplasma negative.

### 2.9 Knockdown and Overexpression of FTSJ3, BOP1, and SREBP1

Cells were seeded at 60% confluence in 6‐well plates for transient overexpression and transfected with the desired plasmids using Lipofectamine 3000 (Thermo Fisher Scientific, Cat#L3000001) at a 3:1 Lipofectamine/DNA ratio. The medium was changed the day after transfection.

For the generation of shRNA constructs targeting FTSJ3, the following sequences were targeted: shFTSJ3‐1: 5'‐AAG GGA ACA TGT TGA AAG ACAT‐3' and shFTSJ3‐2: 5'‐GCA TGT CGC AAG GCT GTG T‐3'. These sequences were cloned into the pLKO.1‐puro plasmid (Addgene, Cat#8453). The pLKO.1‐puro FTSJ3 shRNA plasmids were confirmed by direct sequencing. The pCDH‐puro‐BOP1/SREBP1 plasmids were obtained from Addgene (Cat#46970), as described previously. To produce high‐titer viruses, HEK293T cells were transfected with a three‐plasmid system, including psPAX2 (Addgene, Cat#12260), pMD2.G (Addgene, Cat#12259), and the lentiviral plasmid. Viruses were harvested at 48‐ and 72‐h post‐infection and stored at −80°C until use. To establish stable cell lines, lentivirus‐transduced cells were selected with puromycin (2–3 µg/mL) 48 h post‐infection and single colonies were expanded and validated by Western blotting. For BOP1/SREBP1 protein knockdown, control siRNA (Cat#sc‐37007) (siRNA sequences were listed as the follows: si‐BOP1‐1, 5′‐CCA CAA GAT GCA CGT ACC T‐3′; si‐BOP1‐2, 5′‐TGG AGT GGT ACG ATG ACT T‐3′; si‐control, 5′‐TTC TCC GAA CGT GTC ACG T‐3′.)and si‐HSPA5 RNA (Cat#sc‐29226) (The siRNA sequences are as follows: human HSPA5, 5’‐ AGU GUU GGA AGA UUC UGA U‐3’ and 5’‐AUC AGA AUC UUC CAA CAC U‐3’)were purchased from Santa Cruz and transfected into cells using the siRNA transfection reagent (Cat#sc‐29528) according to the manufacturer's instructions.

### 2.10 FAO Analysis

The extracellular flux analyzer XFe96 was used to analyze FAO metabolism in H520 and SKMES. A total of 2.0 × 10^5^ cells were incubated with substrate restricted medium and inoculated in FAO assay medium (KHB [111 mM NaCl, 4.7 mM KCl, 1.25 mM CaCl_2_, 2 mM MgSO_4_, 1.2 mM NaH_2_PO_4_], supplemented with 2.5 mM glucose, 0.5 mM L carnitine, and 5 mM HEPES). BSA or palmitate BSA FAO was immediately added to the well and the FAO was measured at 15 min.

### 2.11 Co‐Immunoprecipitation

For co‐immunoprecipitation, 50 µL of A/G protein beads were inhaled into 400 µL of IP buffer and rinse twice. The primary antibody was added in 400 µL IP buffer and incubated at 4°C for 2 h. Finally, the beads were precipitated and western blot analysis was performed on the supernatant.

### 2.12 CCK8

Cells were seeded in a 96‐well plate at a density of 5000 cells per well. After 12, 24, and 48 h of treatment, 10% CCK8 reaction solution was added into the cells, incubated for 30 min, and the absorbance was measured at 450 nm.

### 2.13 Transwell

LUSC cells were seeded into the upper chamber of a Transwell insert in serum‐free medium, while the lower was filled with 800 µl of complete medium containing 10% FBS as a chemotactic agent. After one day of cultivation, cells that had migrated to the lower surface of the membrane were fixed and stained with 0.5% crystal violet and calculated.

### 2.14 EdU

Following treatment, the cells were incubated with EdU reagent (10 µm; Beyotime Biotechnology)at 37°C for 2 hours. Incorporated EdU was detected using an Alexa Fluor 488 azide (green fluorescence), and nuclei were counterstained with Hoechst 33342 (blue fluorescence). The percentage of proliferating cells was calculated as the ratio of EdU‐positive cells to total Hoechst 33342‐positive cells.

### 2.15 TUNEL Assay

The tissues were embedded in paraffin or freeze sections and attached to glass slides, or cultured cells were fixed in paraformaldehyde (4%). The sample treated with Triton X‐100 (0.1%–0.5%) or proteinase K, covered with a mixture of TdT enzyme and labeled dUTP, and incubated at 37°C in the dark for 30–60 minutes. Samples without TdT enzyme or pretreated with DNase were used as positive controls. The cells and tissues were harvested and stained with membrane associated protein V‐FITC and propidium iodide after treatment (Vazyme, Nanjing, China). Photos were taken with a fluorescence microscope to observe the fluorescence intensity. These data were detected by flow cytometry (Beckman Coulter, USA).

### 2.16 Western Blot

Total protein was extracted using RIPA lysis buffer (Beyotime, P0013B), separated by 10% SDS‐PAGE (NCM Biotech, P2012), and transferred onto PVDF membranes. After blocking with 5% non‐fat milk in TBST for 1 h at room temperature, membranes were incubated overnight at 4°C with the following primary antibodies (all diluted 1:1000): GAPDH (ab157156, Abcam), FTSJ3 (50607S, Cell Signaling Technology), BOP1 (ab86653, Abcam), DRP1 (ab184247, Abcam), SUMO1 (ab32058, Abcam), SUMO2/3 (ab81371, Abcam), CPT1A (ab128568, Abcam), FASN (ab207369, Abcam), ACLY (67166‐1‐Ig, Proteintech), SCD (sc‐515875, Santa Cruz Biotechnology), SREBP1 (sc‐366, Santa Cruz Biotechnology), and ACC1 (A300‐986A, Thermo Fisher Scientific). After washing with TBST, membranes were incubated with HRP‐conjugated goat anti‐rabbit IgG (1:5000, 31460, Invitrogen) for 30 min at room temperature. Protein bands were visualized by enhanced chemiluminescence (ECL) and analyzed using an Alliance Q9 Advanced imaging system (UVITEC, UK).

### 2.17 Immunohistochemistry

LUSC tumor tissues and adjacent non‐cancerous tissues were fixed in 10% neutral buffered formalin for 24 h at room temperature, washed with Dulbecco's PBS (D‐PBS), and transferred to 70% ethanol. FFPE sections (5 µm) were deparaffinized, rehydrated, and subjected to heat‐induced antigen retrieval by boiling in 0.01 M citrate buffer (pH 6.0) using a 2100 antigen retriever for 20 min. Endogenous peroxidase activity was quenched with 3% H_2_O_2_ for 5 min at room temperature. Sections were blocked with 2.5% goat serum in TBS‐T for 1 h at room temperature and incubated overnight at 4 °C with primary antibodies against FTSJ3 (1:600, 50607S, Cell Signaling Technology) or BOP1 (1:500, sc‐28383, Santa Cruz Biotechnology). HRP‐conjugated secondary antibodies (Vector Laboratories) were applied at 1:200 in TBS‐T for 30 min at room temperature, followed by DAB staining (SK‐4100, Vector Laboratories). Images were acquired using a DFC7000 T digital camera (Leica Microsystems) mounted on an inverted microscope. Digital scanning and quantification were performed with an Aperio GT 450 scanner (Leica Biosystems), and IHC scores were calculated as the product of the percentage of positive cells and staining intensity using Fiji software.

### 2.18 Immunofluorescence

Immunofluorescence staining was performed on human LUSC tissue sections using primary antibodies from three different host species: FTSJ3 (1:200, rabbit, ab186009, Abcam), BOP1 (1:200, mouse, sc‐28383, Santa Cruz Biotechnology), and HSPA5 (1:200, goat, sc‐10586, Santa Cruz Biotechnology). After blocking with 5% normal donkey serum in PBST (0.3% Triton X‐100) for 1 h at room temperature, sections were incubated with the primary antibody cocktail overnight at 4 °C. Species‐specific secondary antibodies were subsequently applied at 1:1000: Alexa Fluor Plus 488–conjugated goat anti‐rabbit IgG (A32731, Thermo Fisher Scientific) for FTSJ3, Alexa Fluor Plus 594–conjugated goat anti‐mouse IgG (A32742, Thermo Fisher Scientific) for BOP1, and Alexa Fluor Plus 647–conjugated donkey anti‐goat IgG (A21447, Thermo Fisher Scientific) for HSPA5. Slides were mounted with DAPI‐containing fluorescence mounting medium (ab104139, Abcam) and examined under a confocal or widefield fluorescence microscope (Leica Microsystems) equipped with a DFC7000 T camera. Images were acquired by sequential scanning to avoid fluorophore cross‐talk.

### 2.19 Tumor Volume In Vivo

The tumor volume of mice was recorded every two days using the vernier caliper, and the measurement of tumor volume was calculated using the formula V = ab2/2.

### 2.20 Hematoxylin and Eosin (H&E) Staining

Tumor tissues were fixed in 10% paraformaldehyde and longitudinally embedded in paraffin. Tissue sections (3–5 µm thick) were stained with H&E and observed under an orthogonal microscope (Nikon Eclipse E100; Nikon, Tokyo, Japan). Images were captured with a Nikon DS‐U3 camera system (Nikon, Tokyo, Japan).

### 2.21 2′‐O‐methylation Sequencing (Nm‐seq)

Tumor tissues and paired adjacent non‐tumor tissues were collected from two patients with lung squamous cell carcinoma (LUSC). Total RNA was extracted using TRIzol reagent. Briefly, equal amounts of total RNA were subjected to rRNA depletion or mRNA enrichment, followed by random fragmentation. Based on the relative resistance of 2’‐O‐methylation sites to alkaline hydrolysis, the RNA fragments were specifically treated under alkaline conditions, and sequencing libraries were subsequently constructed. The libraries underwent end repair, adapter ligation, reverse transcription, and PCR amplification, followed by quality control assessment. Qualified libraries were subjected to high‐throughput sequencing on the Illumina platform. Raw sequencing data were quality‐controlled, and low‐quality reads together with adapter sequences were removed. 2’‐O‐methylation sites were identified by analyzing alkaline‐cleavage protection signals or specific termination signals across transcripts, and the differences in 2’‐O‐methylation levels between LUSC tumor tissues and paired adjacent non‐tumor tissues were compared. Normalized 3’‐end counts for each gene within individual samples were summed, and a signal matrix for tumor and paired normal tissues was constructed. All gene‐level signals were transformed to log2(signal + 1) prior to differential analysis, which was subsequently performed together with functional enrichment analysis.

### 2.22 Reverse Transcription at Low deoxy‐ribonucleoside Triphosphate Concentrations Followed by Polymerase Chain Reaction (RTL‐P)

2’‐O‐methylation levels were detected by the RTL‐P assay. Briefly, specific primers targeting the 2’‐O‐methylation site(s) of HSPA5 were designed. Equal amounts of RNA were subjected to gene‐specific reverse transcription using low (e.g., 0.5 µM) and standard (500 µM) concentrations of deoxyribonucleoside triphosphates (dNTPs), respectively. The resulting cDNA yields from the target regions were then detected by semi‐quantitative PCR. The relative methylation level of the target site was evaluated by calculating the inhibition index, defined as the ratio of PCR product quantities between the low‐dNTP and high‐dNTP groups. Primer sequences are provided in Table S2.

### 2.23 ROS Detection

Mouse tumor tissues and cell suspensions were prepared, centrifuged at 4°C, 1000 rpm, and resuspended in buffer containing 5 µM MitoSOX. The cells were incubated at 37°C in the dark for 10 minutes. After washing, fluorescence intensity was observed and images were captured using a confocal microscope.

### 2.24 Electron Microscopy for Mitochondrial Morphology

Tumor tissues and LUSC cells fixed in 2.5% glutaraldehyde were dehydrated, infiltrated, and embedded to prepare ultrathin sections (60–80 nm) for transmission electron microscopy. Sections were double stained with 3% uranyl acetate and lead citrate (15 minutes each), dried at room temperature overnight, and observed under a transmission electron microscope for image acquisition and analysis.

### 2.25 Mitochondrial Membrane Potential (MMP) Assay

Cells were seeded for 24 hours and treated with the above‐mentioned interventions. TMRE (50 nM) was added to the medium, and the plates were incubated in the dark at 37°C for 30 minutes. Cells were then trypsinized and analyzed by flow cytometry.

### 2.26 ATP Levels

Mouse tumor tissues and cells were homogenized in 200 µL lysis buffer, centrifuged at 4°C, 12000 rpm for 5 minutes, and the supernatant was collected. ATP detection working solution was prepared, and 100 µL was added to the detection wells, incubated at room temperature for 3–5 min. Then, 20 µL of sample or standard was added, mixed quickly with a micropipette, and RLU values were measured. A standard curve was plotted to calculate ATP concentration in samples. Protein concentration was measured to calculate ATP amount per mg of protein, standardizing ATP concentration.

### 2.27 Statistical Analysis

Data are presented as mean ± SD. Student t‐test and One‐way ANOVA were used to compare the average differences between groups. P<0.05 was considered statistically significant.

## 3 Results

### 3.1 FTSJ3 Related to PTMs is Highly Expressed in LUSC

**FIGURE 1:**
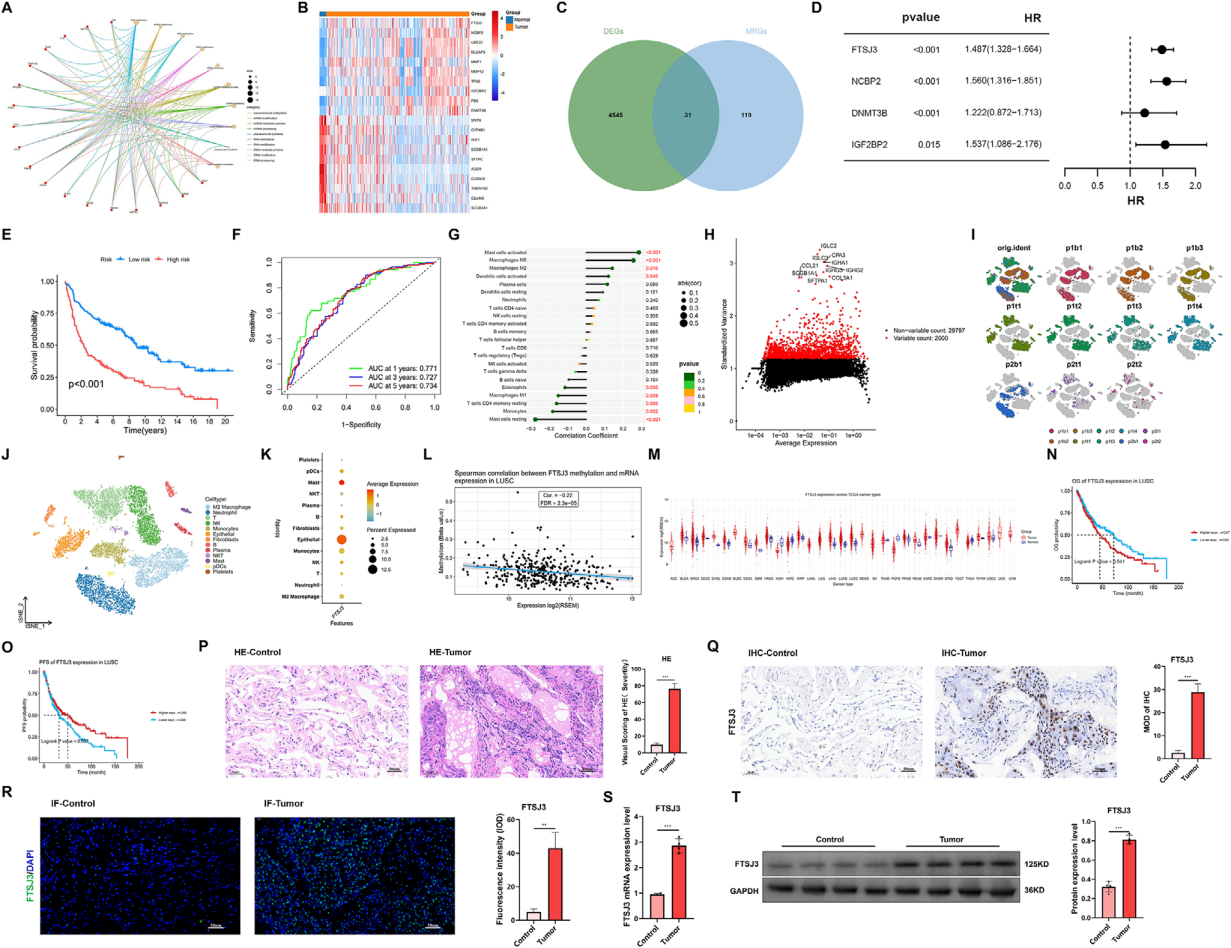
Identification of the RNA methylation modification gene FTSJ3 through bioinformatics analysis. (A) Bp‐cnetplot illustrating the selection of PTM differential genes. Red and yellow dots represent upregulated and downregulated genes, respectively; (B) Heatmap of RNA methylation modifications in LUSC; (C) The Venn diagram of DEGs and MRGs; (D) Forest plot showing the hazard ratios (HR) and 95% confidence intervals (CI) for FTSJ3, NCBP2, DNMT3B, and IGF2BP2; (E) Correlation between methylation and LUSC survival rate; (F) Risk model prediction; (G) Correlation between FTSJ3 and immune cells; (H) Volcano of the top variable genes across various cell populations; (I) Original t‐SNE dimensionality reduction plot; (J) t‐SNE plot showing cell clustering; (K) Dot plot showing the expression of FTSJ3 in different cell types; (L) Spearman correlation analysis of the relationship between methylation modifications and LUSC progression. (M) FTSJ3 expression across TCGA cancer types; (N) FTSJ3 expression across Overall Survival (OS); (O) FTSJ3 expression across Progression‐Free Survival (PFS); (P) H&E staining to observe tumor cell infiltration in tumor and adjacent non‐tumor tissues (left), Visual Scoring of H&E (right); scale bars, 50 µm; (Q) Representative immunohistochemical staining images of FTSJ3 in tumor and corresponding adjacent non‐tumor tissues (left), MOD of IHC (right), scale bars, 50 µm; (R) Representative Immunofluorescence staining images of FTSJ3 in LUSC tumor tissues and non‐tumor tissues (left), Fluorescence intensity of FTSJ3 (right), scale bars, 20 µm; (S) qRT‐PCR analysis of FTSJ3 mRNA levels in LUSC tumor and adjacent non‐tumor tissues; (T) Western blot analysis and quantification of FTSJ3 protein expression in LUSC tumor and adjacent non‐tumor tissues. Experiments were divided into Control group (adjacent non‐tumor tissues) and Tumor group. * compared to Control group, ^**^, *p* < 0.01, ^***^, *p* < 0.001.

To identify clinically relevant RNA modification‐related genes in LUSC, we performed RNA‐seq analysis on 61 LUSC and 14 adjacent non‐cancerous samples from the GSE30219 cohort. Differential expression analysis (|log2 Fold Change|≥0.5, p<0.05) revealed 4,576 DEGs—2,138 upregulated and 2,438 downregulated in tumor tissues (Figure 1A–B). The figure highlights the names of the top five genes with the most significant changes in |log2 fold change|. A literature search identified a total of 150 epigenetic genes that regulate methylation, 31 of which overlapped with the LUSC DEGs (Figure 1C).

Using univariate Cox regression, LASSO, and multivariate Cox regression, we identified four core genes, *DNMT3B, NCBP2, FTSJ3*, and *IGF2BP2*, as risk factors (HR>1) (Figure 1D). A cellular methylation‐related signature for survival prediction was constructed using these four genes via LASSO‐Cox regression. A 4‐gene signature was constructed according to the optimum λ value. The risk score of every patient was then calculated and stratified into low‐ and high‐risk groups according to the median value of the risk score. Kaplan‐Meier survival analysis demonstrated that patients in the high‐risk group had a significantly shorter overall survival (OS) compared with those in the low‐risk group (Figure 1E, log‐rank p < 0.0001). Time‐dependent receiver operating characteristic analysis was performed, and the areas under the curve for 1‐, 3‐, and 5‐year OS were 0.771, 0.727, and 0.734, respectively (Figure 1F). qRT‐PCR results showed that, compared with the control group, the mRNA levels of DNMT3B, NCBP2, FTSJ3, and IGF2BP2 were significantly elevated in the tumor tissues (Figure S1). The Wilcoxon test was employed to assess the differences in 22 types of immune cells between high‐ and low‐risk groups in the GSE30219 training set. The results indicated that one type of immune cell, M2 macrophage, showed significant differences between the high‐risk groups (p < 0.05) and was thus defined as a differential immune cell. Spearman correlation analysis was conducted using the “psych” package to evaluate the relationship between each prognostic gene and the differential immune cell. The analysis revealed a significant positive correlation between the prognostic gene FTSJ3 and M2 macrophage (ρ = 0.14, p < 0.05) (Figure 1G).

We also performed scRNA‐seq analysis on tumor tissue samples from two patients obtained from the GSE127465 dataset. We identified the top highly variable secretory genes (variable count: 2000) and examined their expression patterns across different cell types. We classified the cells into 13 cell types based on the expression profiles of representative marker genes, including M2 macrophage, neutrophil, T, NK, monocytes, epithelial, fibroblasts, B, plasma, NKT, mast, pDCs and platelets (Figure 1H–J). At the same time, we found that FTSJ3 is highly expressed in tumor cells (Figure 1K). FTSJ3 expression is positively associated with LUSC progression, whereas its promoter methylation level is negatively correlated with tumor progression (Figure 1L). Moreover, high expression of FTSJ3 in tumor tissues is significantly associated with poor patient prognosis, including both OS and progression‐free survival (Figure 1M–O). Subsequently, we collected tumor tissues and paired adjacent non‐tumor tissues from patients with LUSC to evaluate FTSJ3 expression. As shown in Figure 1P, compared to adjacent tissues, H&E staining results indicated a marked increase in tumor cell infiltration in LUSC tissues. Figure 1Q demonstrates a significant increase in FTSJ3 expression in tumor tissues compared to adjacent tissues, with further fluorescence analysis confirming the localization of FTSJ3 within tumor cells (Figure 1R). qPCR and western blot results also indicated a significant upregulation of FTSJ3 mRNA and protein expression in tumor tissues compared to adjacent tissues (Figure 1S,T). These data establish FTSJ3 as a prognostically unfavorable epigenetic regulator that is highly expressed in LUSC, implicating it as a potential driver of disease progression.

### 3.2 FTSJ3 Promotes LUSC Progression

**FIGURE 2:**
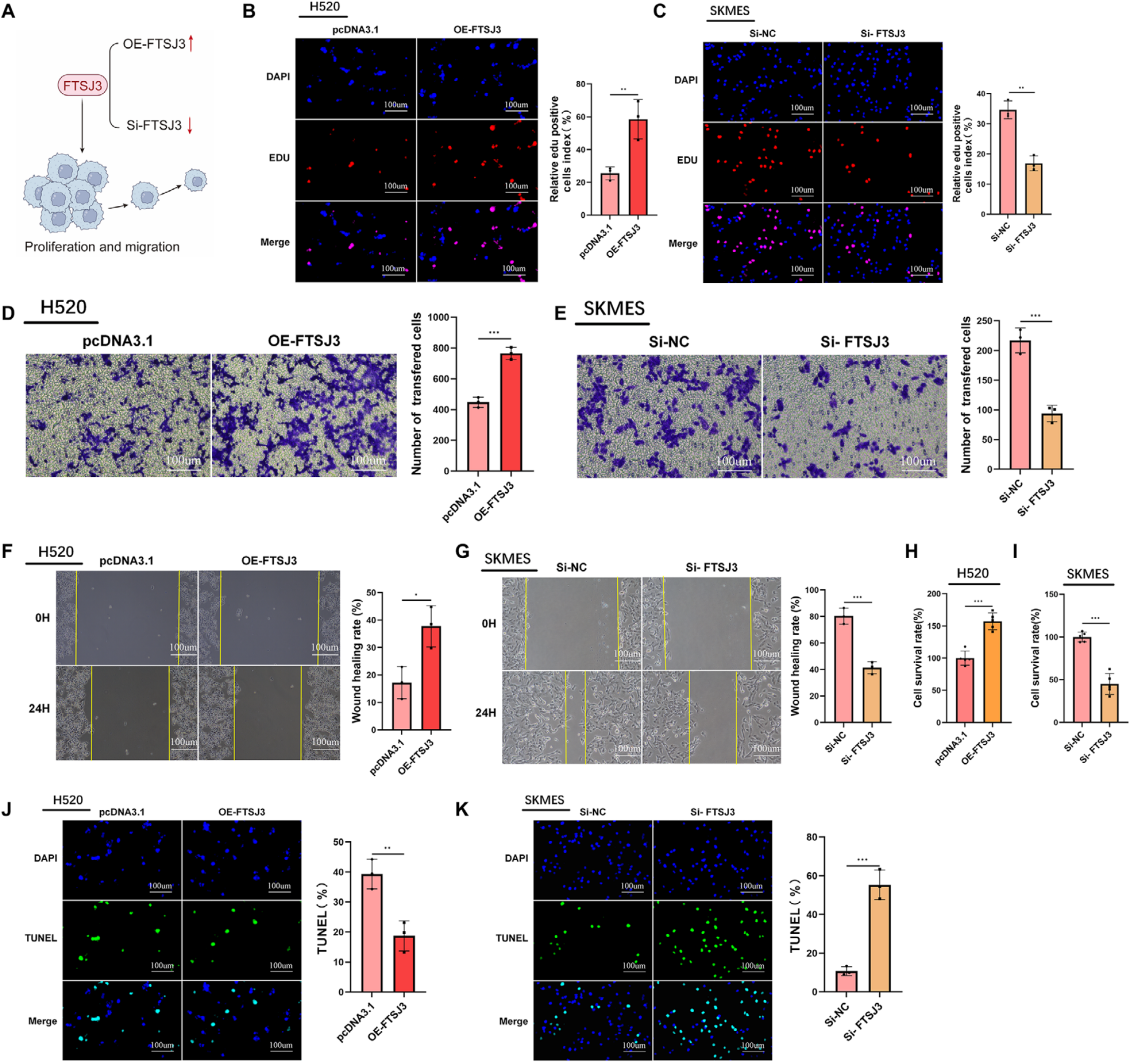
FTSJ3 promotes proliferation and migration of LUSC cells. (A) Flowchart of the experimental procedure: overexpression or knockdown of FTSJ3 in lung squamous cell carcinoma cell lines; (B) EdU assay showing proliferation of H520 cells following FTSJ3 overexpression; (C) EdU assay showing proliferation of SKMES cells following FTSJ3 knockdown; (D) Transwell assay assessing overexpression of FTSJ3 in H520 cells on tumor cell invasion; (E) Transwell assay assessing knockdown of FTSJ3 in SKMES cells on tumor cell invasion; (F) Wound healing assay showing migration of H520 cells following FTSJ3 overexpression; (G) Wound healing assay showing migration of SKMES cells following FTSJ3 knockdown; (H) CCK8 assay showing viability of H520 cells following FTSJ3 overexpression; (I) CCK8 assay showing viability of SKMES cells following FTSJ3 knockdown; (J) TUNEL staining showing apoptosis of H520 cells following FTSJ3 overexpression; (K) TUNEL staining showing apoptosis of SKMES cells following FTSJ3 knockdown. Experiments were divided into Si‐NC group, Si‐FTSJ3 group, pcDNA3.1 group, and OE‐FTSJ3 group. scale bars, 100 µm; * Compared to the empty vector group, ^*^, *p* < 0.05, ^**^, *p* < 0.01, ^***^, *p* < 0.001.

Given its association with poor prognosis, we next investigated whether FTSJ3 functionally drives malignant phenotypes by performing in vitro experiments in the LUSC cell lines H520 and SK‐MES. Transient transfection was conducted to overexpress FTSJ3 in H520 cells or to knock down FTSJ3 in SK‐MES cells (Figure 2A). Subsequent assessments of cell viability, proliferation, migration, and invasion were conducted. As depicted in Figure 2, compared to the empty vector control group, overexpression of FTSJ3 significantly enhanced LUSC cell viability, proliferation, migration, and invasion, while apoptosis was inhibited (Figure 2B,D,F,H,J). Conversely, FTSJ3 knockdown resulted in reduced cell viability, proliferation, migration, and invasion compared to the negative control group (Figure 2C,E,G,I,K). Thus, FTSJ3 is both necessary and sufficient to drive proliferative and invasive growth of LUSC cells in vitro.

### 3.3 FTSJ3 Regulates the 2′‐O‐Methylation of HSPA5 mRNA

**FIGURE 3:**
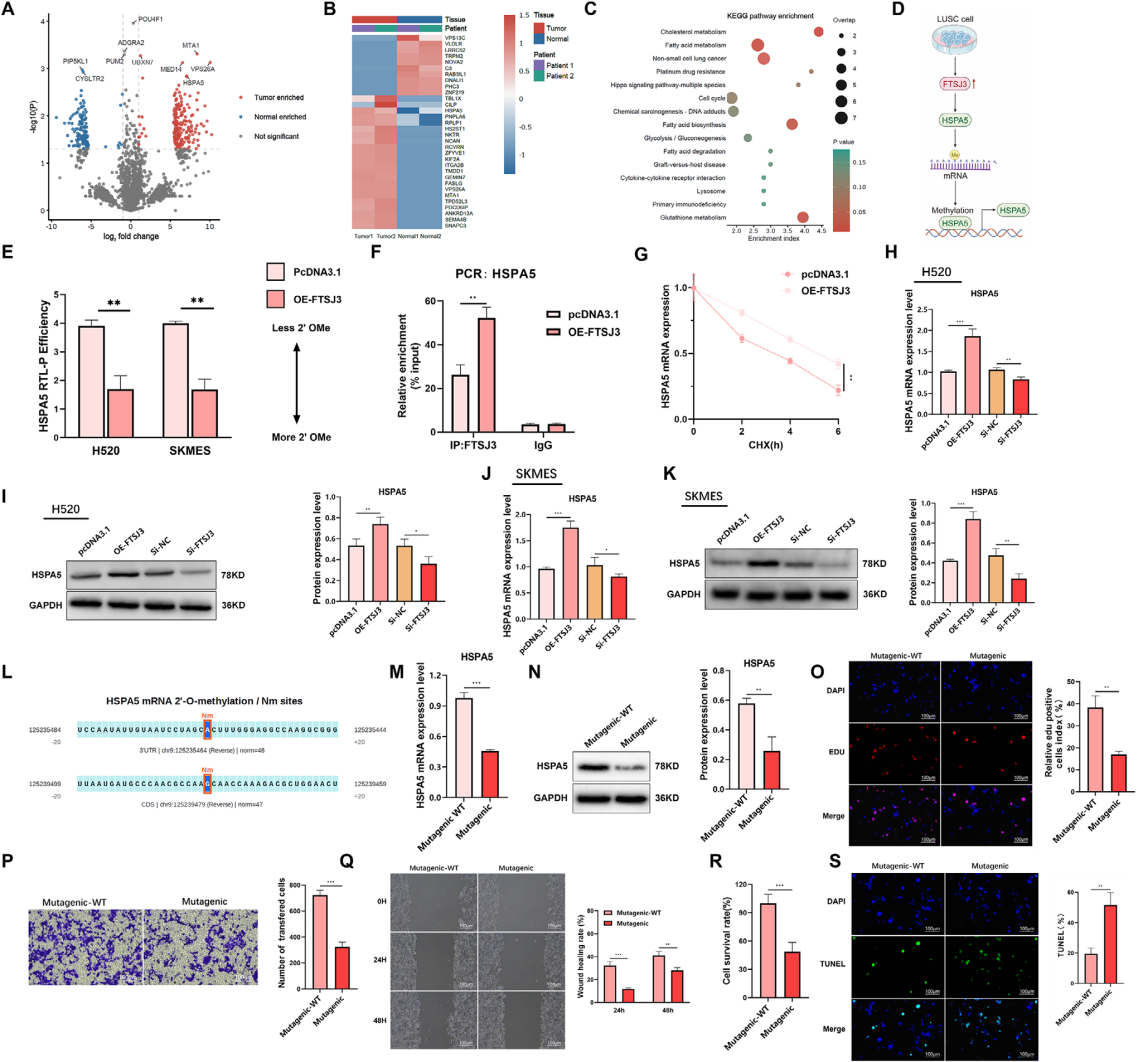
FTSJ3 regulates HSPA5 mRNA 2’‐O‐methylation to promote proliferation and migration of LUSC cells. (A) Volcano plot of differentially expressed 2’‐O‐methylation genes in LUSC; (B) Heatmap of differentially expressed 2’‐O‐methylation genes in LUSC; (C) KEGG pathway analysis of differentially expressed 2’‐O‐methylation genes; F. Flowchart of the experimental procedure: FTSJ3 regulates the methylation of HSPA5 in tumor cells; (E) The 2’‐O‐methylation level of HSPA5 mRNA was assessed by RTL‐P assay. (F) RIP assays showing the association of FTSJ3 with HSPA5 upon FTSJ3 overexpression. (G) qRT‐PCR analysis of HSPA5 mRNA levels after treatment with Actinomycin D at the indicated time points in H520 cells. (H) qRT‐PCR analysis of HSPA5 mRNA levels in H520 cells following FTSJ3 intervention; (I) Western blot analysis and quantification of HSPA5 protein expression in H520 cells following FTSJ3 intervention; (J) qRT‐PCR analysis of HSPA5 mRNA levels in SKMES cells following FTSJ3 intervention; (K) Western blot analysis and quantification of HSPA5 protein expression in SKMES cells following FTSJ3 intervention; (L) Illustration of HSPA5 mRNA 2’‐O‐methylation site mutation; (M) qRT‐PCR quantitative analysis of HSPA5 mRNA expression after 2’‐O‐methylation site mutation; (N) Western blot analysis and quantification of HSPA5 protein expression after site mutation; (O) EdU assay assessing the impact of mutation on tumor cell proliferation; (P) Transwell assay assessing the impact of mutation on tumor cell invasion; (Q) Wound healing assay assessing the impact of mutation on tumor cell migration; (R) CCK8 assay assessing tumor cell viability following mutation of 2’‐O‐methylation site mutation; (S) TUNEL assay assessing the impact of mutation on tumor cell apoptosis. Scale bars, 100 µm. Experiments were divided into a mutation group and a mutation control group. * Compared to the mutation control group, ^*^, *p* < 0.05, ^**^, *p* < 0.01, ^***^, *p* < 0.001.

As an RNA methyltransferase [31], we hypothesized that FTSJ3 exerts its oncogenic effects by modifying key downstream targets. To investigate the mechanism of 2'‐O‐methylation, we performed sequencing on tumor tissues and matched normal lung tissues. We identified 185 upregulated and 128 downregulated genes, with a significant increase in the 2'‐O‐methylation level of HSPA5 mRNA (Figure 3A,B). We also observed a significant increase in the expression of HSPA5 in LUSC tumor tissues compared to normal tissues (Figure S2). The KEGG functional enrichment analysis revealed that pathways such as NSCLC, fatty acid metabolism, and cholesterol metabolism were involved (Figure 3C). FTSJ3 regulated the methylation level of HSPA5 mRNA (Figure 3D). RTL‐P analysis revealed that FTSJ3 overexpression led to a significant increase in the 2'‐O‐methylation level of HSPA5 (Figure 3E). Overexpression of FTSJ3 enhanced the interaction between FTSJ3 and HSPA5 protein–RNA complexes and increased the stability of HSPA5 mRNA (Figure 3F,G). Additionally, we found that FTSJ3 overexpression in LUSC cells increased the mRNA and protein levels of HSPA5, whereas FTSJ3 knockdown led to a reduction in the expression levels of HSPA5 (Figure 3H–K). Subsequently, we generated a mutant plasmid in which the putative 2'‐O‐methylation (Nm) site, located at the 21st nucleotide within a 41‐nt transcript region of HSPA5, was disrupted by a point mutation (Figure 3L). Expression of this mutant markedly reduced the mRNA and protein levels of HSPA5 in H520 cells (Figure 3M,N). Mutation of the 2'‐O‐methylation site in HSPA5 resulted in significantly reduced proliferation, viability, and migration of LUSC cells, while apoptosis was notably increased (Figure 3O–S). These results demonstrate that FTSJ3‐catalyzed 2'‐O‐methylation of HSPA5 stabilizes HSPA5 mRNA and is essential for its tumor‐promoting function in LUSC, driving proliferation, migration, and invasion.

### 3.4 FTSJ3 Regulates the SUMOylation of BOP1 via HSPA5 to Promote LUSC Progression

**FIGURE 4:**
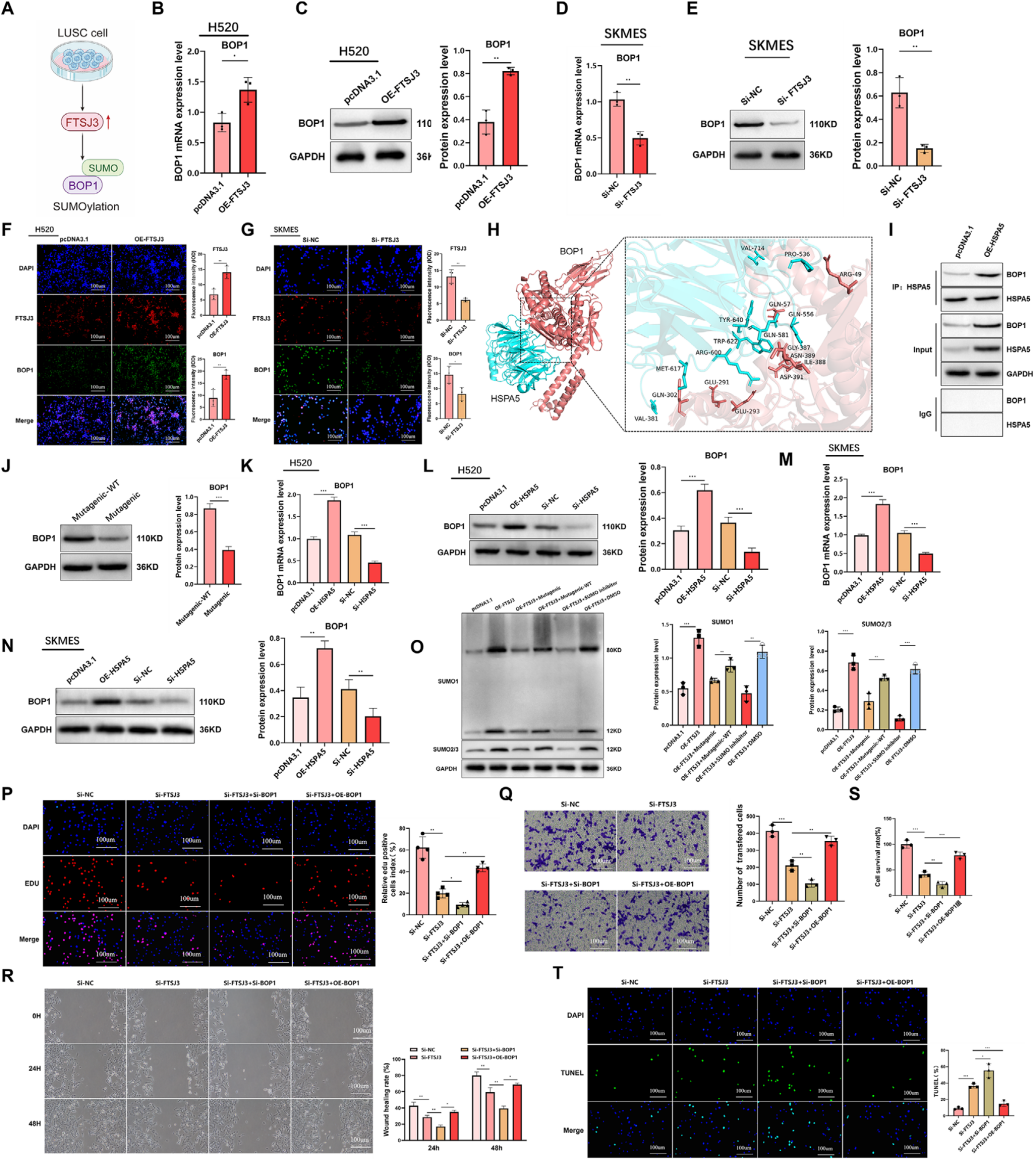
FTSJ3 regulates BOP1 SUMOylation via HSPA5 to promote proliferation and migration of LUSC cells. (A) Flowchart of the experimental procedure: FTSJ3 regulates BOP1 SUMOylation in tumor cells; (B) qRT‐PCR analysis of BOP1 mRNA levels in H520 cells following FTSJ3 intervention; (C) Western blot analysis and quantification of BOP1 protein expression in H520 cells following FTSJ3 intervention; (D) qRT‐PCR analysis of BOP1 mRNA levels in SKMES cells following FTSJ3 intervention; (E) Western blot analysis and quantification of BOP1 protein expression in SKMES cells following FTSJ3 intervention; (F) Representative Immunofluorescence staining images of FTSJ3 and BOP1 in H520 cells following FTSJ3 intervention (left), Fluorescence intensity of FTSJ3 and BOP1 (right), scale bars, 100 µm; (G) Representative Immunofluorescence staining images of FTSJ3 and BOP1 in SKMES cells following FTSJ3 intervention (left), Fluorescence intensity of FTSJ3 and BOP1 (right), scale bars, 100 µm; (H) Schematic diagram showing the binding of protein HSPA5 with BOP1. (I) The levels of BOP1 on HSPA5 were assessed by Co‐IP assays. (J) Western blot analysis and quantification of BOP1 protein expression after site mutation; (K) qRT‐PCR analysis of BOP1 mRNA levels in H520 cells following HSPA5 intervention; (L) Western blot analysis and quantification of BOP1 protein expression in H520 cells following HSPA5 intervention; (M) qRT‐PCR analysis of BOP1 mRNA levels in SKMES cells following HSPA5 intervention; (N) Western blot analysis and quantification of BOP1 protein expression in SKMES cells following HSPA5 intervention; (O) Western blot analysis and quantification of SUMO1 and SUMO 2/3 protein expression in H520 cells; (P) EdU assay assessing the impact of FTSJ3 and BOP1 intervention on tumor cell proliferation; (Q) Transwell assay assessing the impact of FTSJ3 and BOP1 intervention on tumor cell invasion; (R) Wound healing assay assessing the impact of FTSJ3 and BOP1 intervention on tumor cell migration; (S) CCK8 assay assessing tumor cell viability following FTSJ3 and BOP1 intervention; (T) TUNEL assay assessing the impact of FTSJ3 and BOP1 intervention on tumor cell apoptosis. scale bars, 100 µm, Experiments were divided into: pcDNA3.1 (overexpression plasmid control) group, OE‐FTSJ3 group (FTSJ3 overexpression group); Si‐NC group (knockdown control group); Si‐FTSJ3 group (FTSJ3 knockdown group); pcDNA3.1 (overexpression plasmid control) group, OE‐ FTSJ3 group (FTSJ3 overexpression group); pcDNA3.1 (overexpression plasmid control) group, OE‐HSPA5 group (HSPA5 overexpression group); Si‐NC group (knockdown control group); Si‐HSPA5 group (HSPA5 knockdown group); Si‐BOP1 group (Si‐BOP1 knockdown group); OE‐BOP1 group (BOP1 overexpression group). * Compared to the negative control group, ^*^, *p* < 0.05, ^**^, *p* < 0.01, ^***^, *p* < 0.001.

BOP1 has been shown to promote the progression of various malignant tumors [32, 33], but its role in LUSC is unknown. We next sought to identify the effector downstream of the FTSJ3‐HSPA5 axis. BOP1 was positively upregulated in LUSC compared with the control group through conducting LUSC transcriptome (Figure S3). We found that FTSJ3 overexpression in LUSC cells increased the mRNA and protein levels of BOP1, whereas FTSJ3 knockdown led to a reduction in the expression levels of BOP1 (Figure 4A–E). Immunofluorescence analysis yielded consistent results, showing that overexpression of FTSJ3 significantly increased BOP1 expression in H520 cells, while FTSJ3 knockdown led to a reduction in BOP1 levels in SK‐MES cells (Figure 4F,G).

We performed protein–protein interaction (PPI) network analysis based on the LUSC transcriptomic data, which showed that BOP1 is located at the center of the network and interacts with HSPA5 (Figure S4A). Our prediction indicated a potential interaction between HSPA5 and BOP1. Subsequently, Co‐IP assays confirmed that overexpression of HSPA5 resulted in an enhanced binding between HSPA5 and BOP1 (Figure 4H,I). In addition, overexpression of FTSJ3 also enhanced the interaction between HSPA5 and BOP1 (Figure S4B). The HSPA5 mutation reduces the expression level of BOP1 (Figure 4J). Subsequent analysis using qPCR and Western blot revealed that BOP1 expression significantly increased following HSPA5 overexpression, whereas BOP1 expression markedly decreased upon HSPA5 knockdown (Figure 4K–N).

Based on previous studies, it has been confirmed that HSPA5 can regulate ubiquitination modification [34]. Therefore, it is speculated that HSPA5 and SUMO can interact with each other. We found that FTSJ3 could regulate the SUMOylation of BOP1 via HSPA5 (Figure 4O). Overexpression of FTSJ3 resulted in a significant increase in the SUMOylation of BOP1, which was reversed upon HSPA5 mutation or treatment with SUMOylation inhibitors (Figure 4O). Furthermore, Co‐IP assays demonstrated that FTSJ3 overexpression significantly increased the SUMOylation level of BOP1 (Figure S5A). The SUMOylation level of BOP1 was increased upon HSPA5 overexpression and decreased upon HSPA5 knockdown (Figure S5B). These results suggest that FTSJ3 and HSPA5 regulate BOP1 protein stability and expression via SUMOylation. Moreover, knockdown of FTSJ3 resulted in the inhibition of proliferation, migration, and colony formation in LUSC cells. Conversely, overexpression of BOP1 restored these cellular functions. Additionally, simultaneous knockdown of FTSJ3 and BOP1 led to further suppression of proliferation, migration, and colony formation in LUSC cells (Figure 4P–T and Figure S6). Therefore, BOP1 SUMOylation serves as a critical molecular link, transducing the FTSJ3‐HSPA5 signal to drive LUSC progression. Of note, manipulation of FTSJ3 or HSPA5 also altered global SUMOylation levels in whole‐cell lysates (input) (Figure S5A,B), suggesting that the FTSJ3‐HSPA5 axis may exert broader regulatory effects on the cellular SUMOylation machinery beyond BOP1 alone [35].

### 3.5 FTSJ3 Promotes Mitochondrial Fission and FAO Metabolism Through HSPA5/BOP1/DRP1/SREBP1 Axis

Given that BOP1 is a core component essential for ribosome biogenesis [36, 37], we investigated how its SUMOylation contributes to metabolic reprogramming. We observed that BOP1 overexpression led to increased SREBP1 expression (Figure S7A,B). As active ribosome biogenesis is frequently coupled with SREBP1‐driven lipogenesis to support membrane biogenesis in rapidly proliferating cells [38], this positive correlation likely reflects an indirect, ribosome biogenesis‐dependent metabolic coordination rather than direct transcriptional activation. Subsequently, BOP1 was overexpressed while SREBP1 was simultaneously silenced in H520 cells. We observed a marked reduction in lipid accumulation, accompanied by decreased levels of total cholesterol (TC) and triglycerides (TG) (Figure S7C–E). Furthermore, the activity for FAO was significantly decreased (Figure S7F). The mRNA and protein levels of metabolic marker genes FASN, ACC, ACLY, and SCD were also decreased (Figure S7G–K). Co‐overexpression of BOP1 and SREBP1 in H520 cells reversed the previously observed effect in Figure S7. We also found that downregulation of BOP1 in H520 and SK‐MES cells reduced the expression of SREBP1 (Figure S8A,B). Concurrently, lipid accumulation decreased, along with reduced levels of total cholesterol (TC) and triglycerides (TG), as well as diminished fatty acid oxidation activity (Figure S8C–J). In addition, the protein expression levels of metabolic markers, including FASN, ACC, ACLY, and SCD, were significantly decreased (Figure S8K,L). Notably, these effects were reversed by SREBP1 overexpression in Figure S8. These findings suggest that increased BOP1 expression can promote FAO metabolism through elevating the expression of SREBP1 in H520 cells.

**FIGURE 5:**
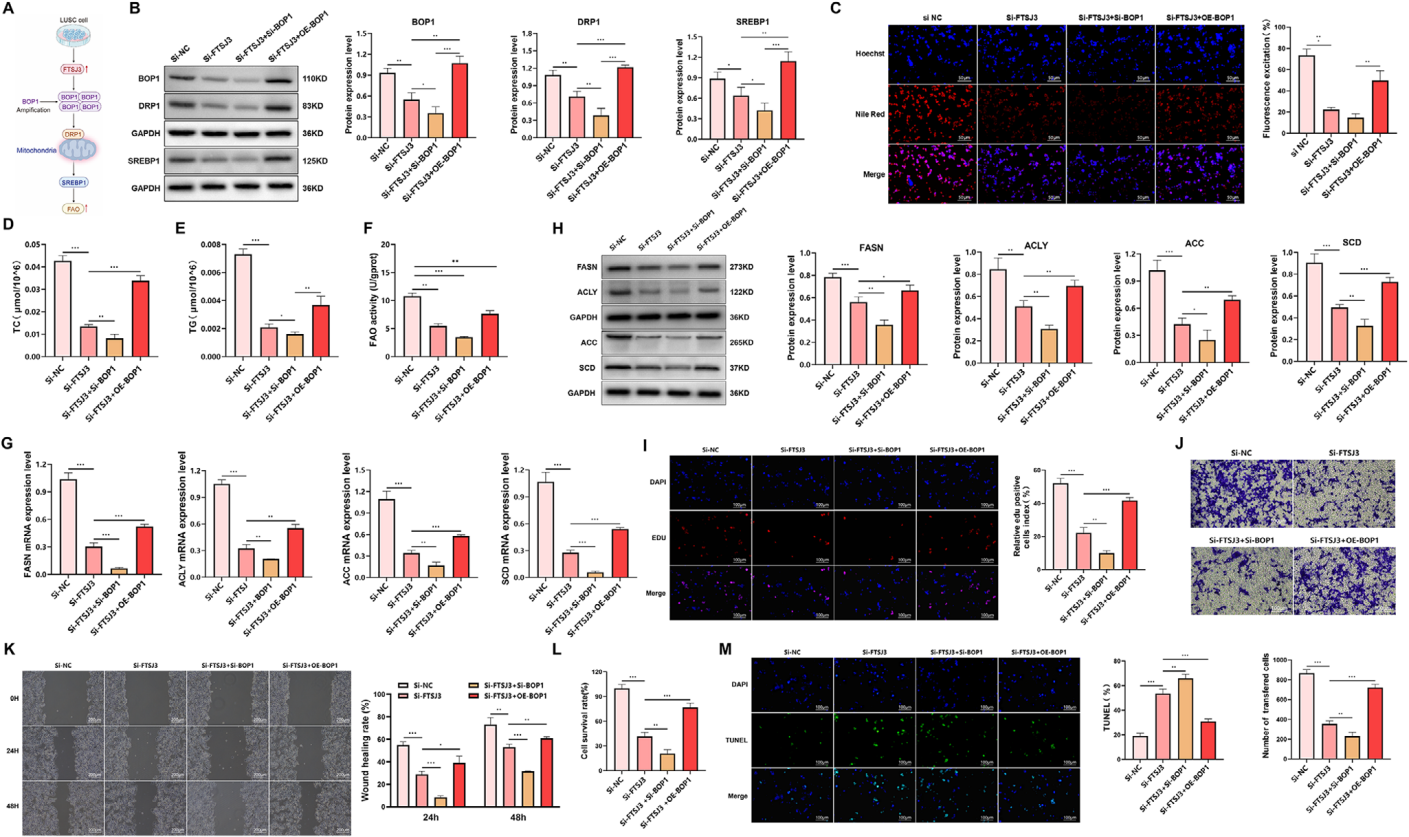
FTSJ3 regulates FAO metabolism via BOP1 to promote proliferation and migration of LUSC cells. SKMES cells were treated with Si‐BOP1 or OE‐BOP1 after treated with Si‐FTSJ3. (A) Flowchart of the experimental procedure: FTSJ3 regulates FAO metabolism via BOP1 in tumor cells; (B) Western blot analysis and quantification of BOP1, DRP1 and SREBP1 protein expression; (C) Nile red level detection using a kit (left), quantification of Fluorescence excitation (right), scale bars, 50 µm; (D) TC content detection using a kit; (E) TG content detection using a kit; (F) FAO activity detection using a kit; (G) qRT‐PCR analysis of FASN, ACC, ACLY, and SCD mRNA levels; (H) Western blot analysis and quantification of FASN, ACC, ACLY, and SCD protein expression; (I) EdU assay assessing on tumor cell proliferation; (J) Transwell assay assessing on tumor cell invasion; (K) Wound healing assay on tumor cell migration; (L) CCK8 assay assessing tumor cell viability; (M) TUNEL assay on tumor cell apoptosis; scale bars, 100 µm, Experiments were divided into knockdown control group (Si‐NC), FTSJ3 knockdown group (Si‐FTSJ3), FTSJ3 overexpression combined with BOP1 knockdown group (OE‐FTSJ3+Si‐BOP1), and FTSJ3 knockdown combined with BOP1 overexpression group (Si‐FTSJ3+OE‐BOP1).* Compared to the Si‐NC or Si‐FTSJ3 group, ^*^, *p* < 0.05, ^**^, *p* < 0.01, ^***^, *p* < 0.001.

In SK‐MES cells, FTSJ3 knockdown downregulated the protein levels of BOP1, DRP1, and SREBP1 (Figure 5A,B). Moreover, silencing FTSJ3 reduced lipid accumulation, which was accompanied by decreases in TC and TG levels, as well as decreased FAO activity (Figure 5C–F). Consistently, the mRNA and protein levels of FASN, ACLY, ACC, and SCD were also reduced (Figure 5G,H). Furthermore, overexpression of BOP1 in FTSJ3‐silenced SK‐MES cells effectively reversed the aforementioned effects in Figure 5. In addition, BOP1 overexpression also rescued the suppressed proliferation, migration, and invasion caused by FTSJ3 knockdown in SK‐MES cells (Figure 5I–M).

**FIGURE 6:**
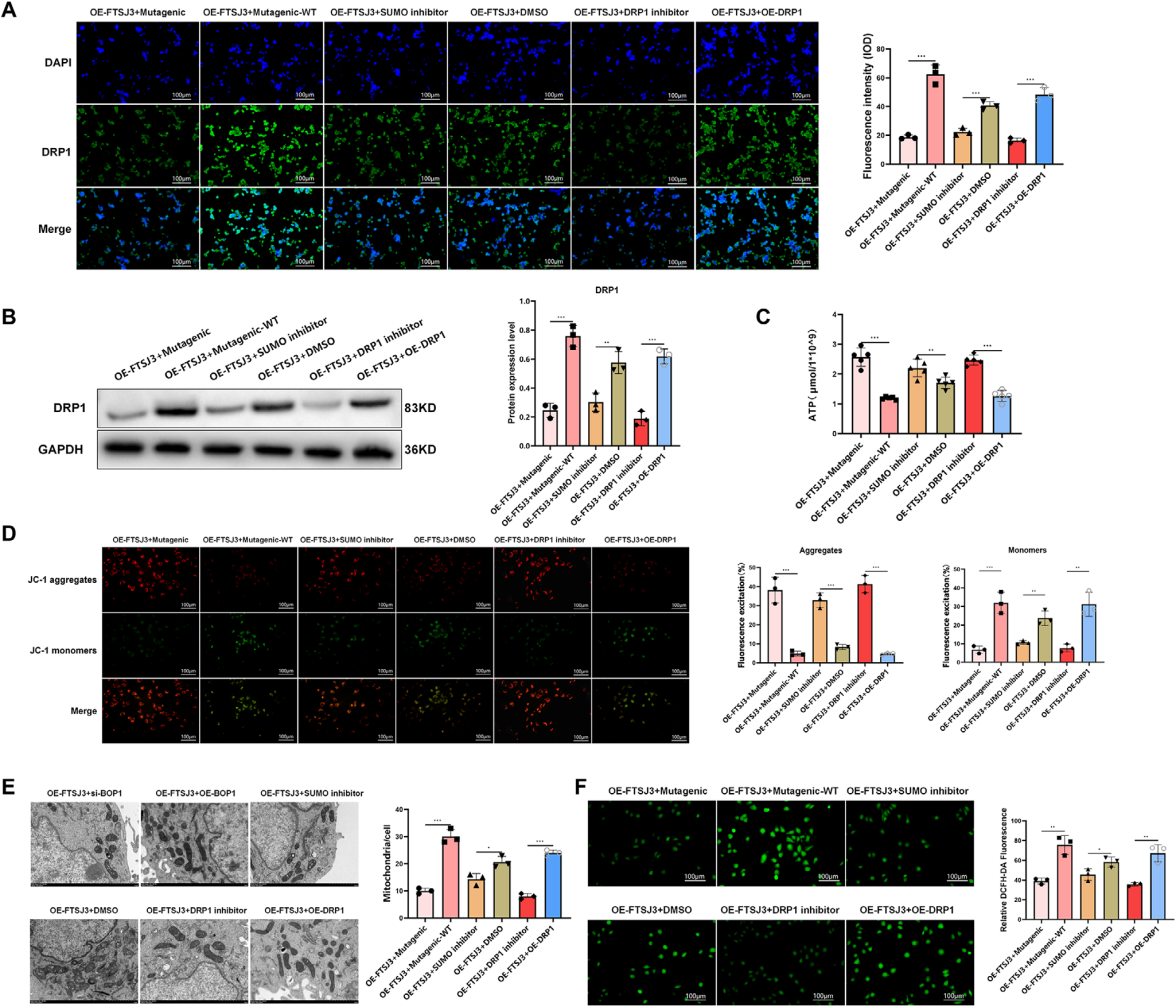
FTSJ3 regulates mitochondrial fission via HSPA5/BOP1/DRP1 axis in LUSC cells. H520 cells were treated with Mutagenic, Mutagenic‐WT, SUMO inhibitor, DMSO, DRP1 inhibitor, or OE‐DRP1 after treated with OE‐FTSJ3. (A) Representative Immunofluorescence staining images of DRP1(left), Fluorescence intensity of DRP1 (right); (B) Western blot analysis and quantification of DRP1 protein expression; (C) ATP assay assessing mitochondrial function; (D) Representative Immunofluorescence staining images of mitochondrial activity (left), quantification of Aggregates and Monomers (right); (E) Representative TEM images of mitochondrial morphology (left), quantification of Mitochondria (right); (F) Representative Immunofluorescence staining images of ROS (left), quantification of relative DCFH‐DA Fluorescence (right). scale bars, 100 µm, Experiments were divided into OE‐FTSJ3+Mutagenic group, OE‐FTSJ3+Mutagenic‐WT group, OE‐FTSJ3+SUMO inhibitor group, OE‐FTSJ3+DMSO group, OE‐FTSJ3+DRP1 inhibitor group, OE‐FTSJ3+OE‐DRP1 group. * Compared to the OE‐FTSJ3+Mutagenic, OE‐FTSJ3+SUMO inhibitor or OE‐FTSJ3+DRP1 inhibitor group, ^*^, *p* < 0.05, ^**^, *p* < 0.01, ^***^, *p* < 0.001.

After overexpressing FTSJ3, H520 cells were treated with either HSPA5 mutation or WT plasmids, a SUMOylation inhibitor or DMSO, and a DRP1 inhibitor or OE‐DRP1. IF and western blot results revealed that under conditions of HSPA5 mutation, SUMOylation inhibition, or DRP1 inhibition, DRP1 expression levels were decreased (Figure 6A,B). Subsequently, we observed that, compared to the overexpression of FTSJ3, knockdown of HSPA5, BOP1, or DRP1 led to a reduction in ATP levels, restoration of MMP, alleviation of mitochondrial damage, and a decrease in ROS production (Figure 6C–F).

These data delineate a linear FTSJ3‐HSPA5‐BOP1‐DRP1‐SREBP1 axis that underscores the critical role of FTSJ3 in orchestrating lipid metabolism, FAO activity, and tumor progression in LUSC.

### 3.6 FTSJ3 Induced LUSC Progression via Enhancing Mitochondrial Fission and FAO Metabolism in Mice

**FIGURE 7:**
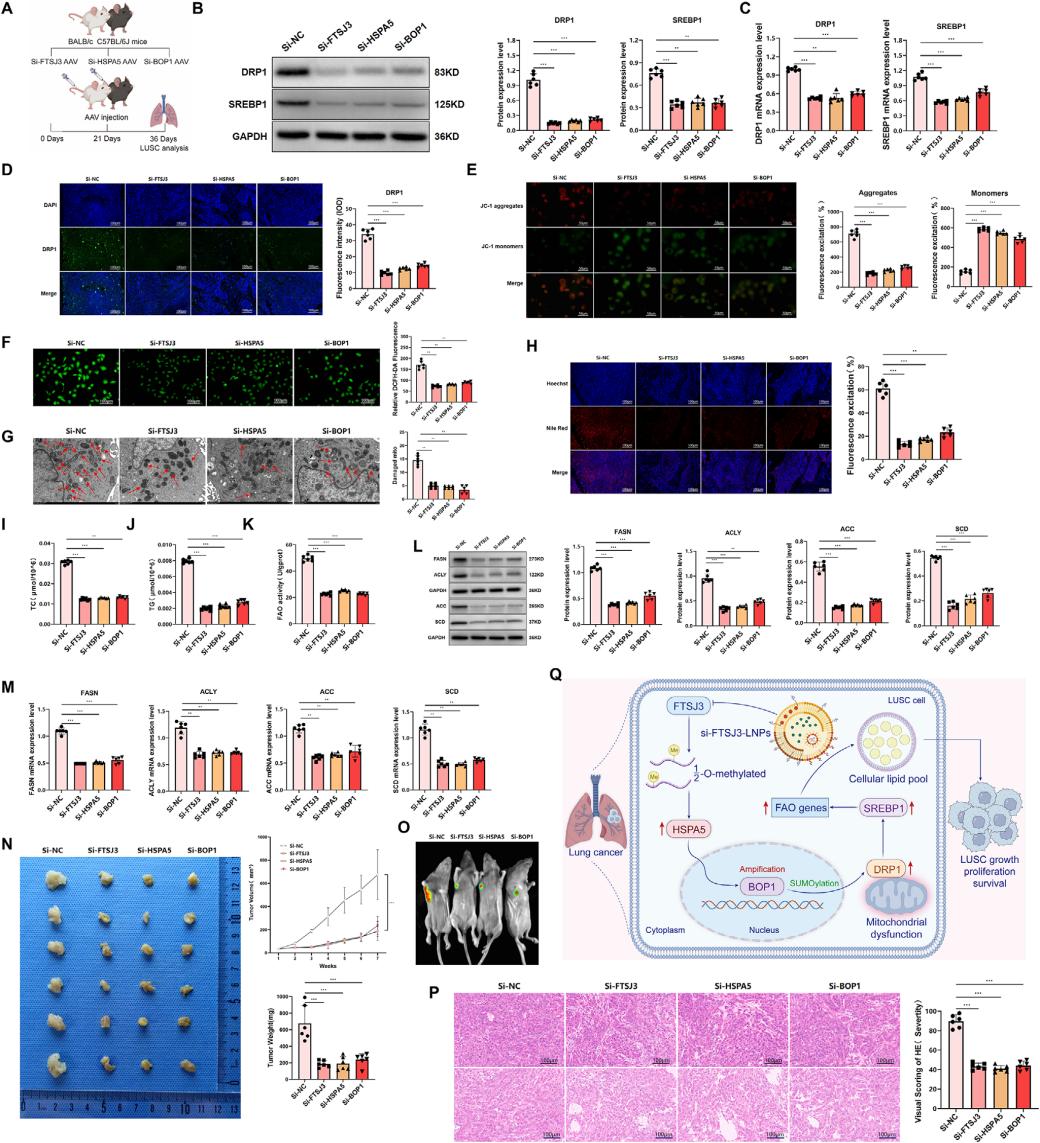
Inhibition of FTSJ3, HSPA5, and BOP1 blocks LUSC proliferation and metastasis by improving mitochondrial fission and FAO metabolism in tumor‐bearing mice. (A) Flowchart of the animal experiment: Utilizing adeno‐associated viruses on tumor‐bearing mice and xenografted mice; (B) Western blot analysis and quantification of DRP1 and SREBP1 protein expression; (C) qRT‐PCR analysis of DRP1 and SREBP1 mRNA levels. (D) Representative Immunofluorescence staining images of DRP1 (left), Fluorescence intensity of DRP1 (right), scale bars, 100 µm; (E) Representative Immunofluorescence staining images of mitochondrial activity (left), quantification of Aggregates and Monomers (right), scale bars, 50 µm; (F) Representative Immunofluorescence staining images of ROS (left), quantification of relative DCFH‐DA Fluorescence (right), scale bars, 100 µm; (G) Representative TEM images of mitochondrial morphology (left), quantification of Mitochondria (right); (H) Nile red level detection using a kit (left), quantification of Fluorescence excitation (right), scale bars, 100 µm; (I) TC content detection using a kit; (J) TG content detection using a kit; (K) FAO activity detection using a kit; (L) Western blot analysis and quantification of FASN, ACLY, ACC, and SCD protein expression; (M) qRT‐PCR analysis of FASN, ACC, ACLY, and SCD mRNA levels; (N) Changes in tumor size (left); Changes in tumor volume and weight (right); (O) In vivo observation of tumor metastasis (P) H&E staining to observe tumor cell infiltration (left), Visual Scoring of H&E (right), scale bars, 100 µm; (Q) Overall flowchart of the study; Experiments were divided into Si‐NC group, Si‐FTSJ3 group, Si‐HSPA5 group, and Si‐BOP1 group (*n* = 6). * Compared to the negative control group, ^*^, *p* < 0.05, ^**^, *p* < 0.01, ^***^, *p* < 0.001.

To validate the physiological relevance of this axis, FAO metabolism and mitochondrial fission‐related indicators were assessed in mouse models with knockdown of FTSJ3, HSPA5, and BOP1 (Figure 7A). Western blot and qRT‐PCR indicated that the mRNA and protein levels of DRP1 and SREBP1 were significantly reduced upon knockdown of FTSJ3, HSPA5, and BOP1, which was consistent with the results of immunofluorescence. (Figure 7B–D). Following JC‐1 staining, knockdown of FTSJ3, HSPA5, and BOP1 was found to reverse the reduction in MMP and the increase in ROS observed in mouse tumor cells (Figure 7E,F). TEM analysis also revealed that inhibition of FTSJ3, HSPA5, and BOP1 expression reversed mitochondrial damage (Figure 7G). We also observed a marked reduction in lipid accumulation, accompanied by decreased levels of TC, TG, and FAO activity, associated with the reduced expression of FTSJ3, HSPA5, and BOP1 (Figure 7H–K). Simultaneous knockdown of FTSJ3, HSPA5, and BOP1 inhibited both the mRNA and protein levels of FASN, ACC, ACLY, and SCD (Figure 7L,M). Moreover, downregulation of FTSJ3, HSPA5, and BOP1 expression resulted in a decrease in tumor volume, weight, and size, as well as reduced tumor cell infiltration (Figure 7N–P).

**FIGURE 8:**
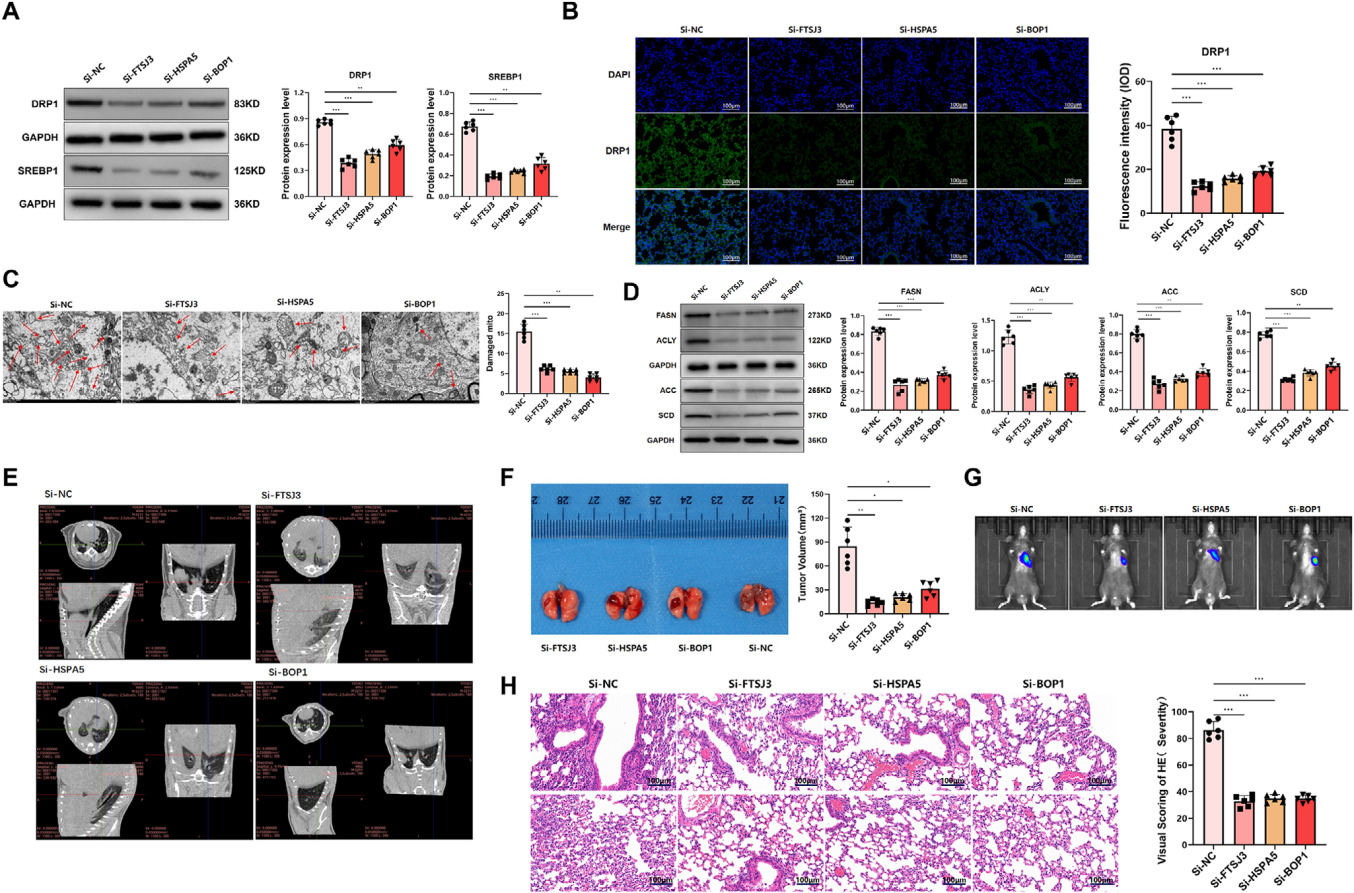
Inhibition of FTSJ3, HSPA5, and BOP1 blocks LUSC proliferation and metastasis in xenografted mice. (A) Western blot analysis and quantification of DRP1 and SREBP1 protein expression; (B) Representative Immunofluorescence staining images of DRP1 (left), Fluorescence intensity of DRP1 (right), scale bars, 100 µm; (C) Representative TEM images of mitochondrial morphology (left), quantification of Mitochondria (right); (D) Western blot analysis and quantification of FASN, ACC, ACLY, and SCD protein expression; (E) CT scan observing tumor changes; (F) Changes in tumor size (left) and weight (right); (G) In vivo imaging observing tumor metastasis; (H) H&E staining to observe tumor cell infiltration (left), Visual Scoring of H&E (right), scale bars, 100 µm; Experiments were divided into Si‐NC group, Si‐FTSJ3 group, Si‐HSPA5 group, and Si‐BOP1 group (*n* = 6). * Compared to the negative control group, ^*^, *p* < 0.05, ^**^, *p* < 0.01, ^***^, *p* < 0.001.

Our experimental findings were further validated in xenograft models. Western blot and immunofluorescence staining indicated that the protein levels of DRP1 and SREBP1 were significantly reduced upon knockdown of FTSJ3, HSPA5, and BOP1 (Figure 8A,B). TEM analysis revealed that the inhibition of the expression of FTSJ3, HSPA5 and BOP1 reversed mitochondrial damage (Figure 8C). Compared to the control group, FASN, ACLY, ACC, and SCD levels were decreased following FTSJ3, HSPA5, and BOP1 knockdown (Figure 8D). Knockdown of FTSJ3, HSPA5, and BOP1 resulted in a significant reduction in LUSC tumor volume and size compared to the control group (Figure 8E,F). Additionally, there was a marked decrease in tumor cell infiltration (Figure 8H), as well as reduced tumor invasiveness. In vivo observations of tumor metastasis, as shown in Figure 8G, demonstrated that knockdown of FTSJ3, HSPA5, and BOP1 significantly reduced tumor metastasis. These in vivo results confirm that the FTSJ3‐HSPA5‐BOP1 axis is a key driver of mitochondrial fission, FAO, and tumor progression in LUSC.

### 3.7 Inhibitory Effect of Liposomes Loaded With FTSJ3 Inhibitor on LUSC

**FIGURE 9:**
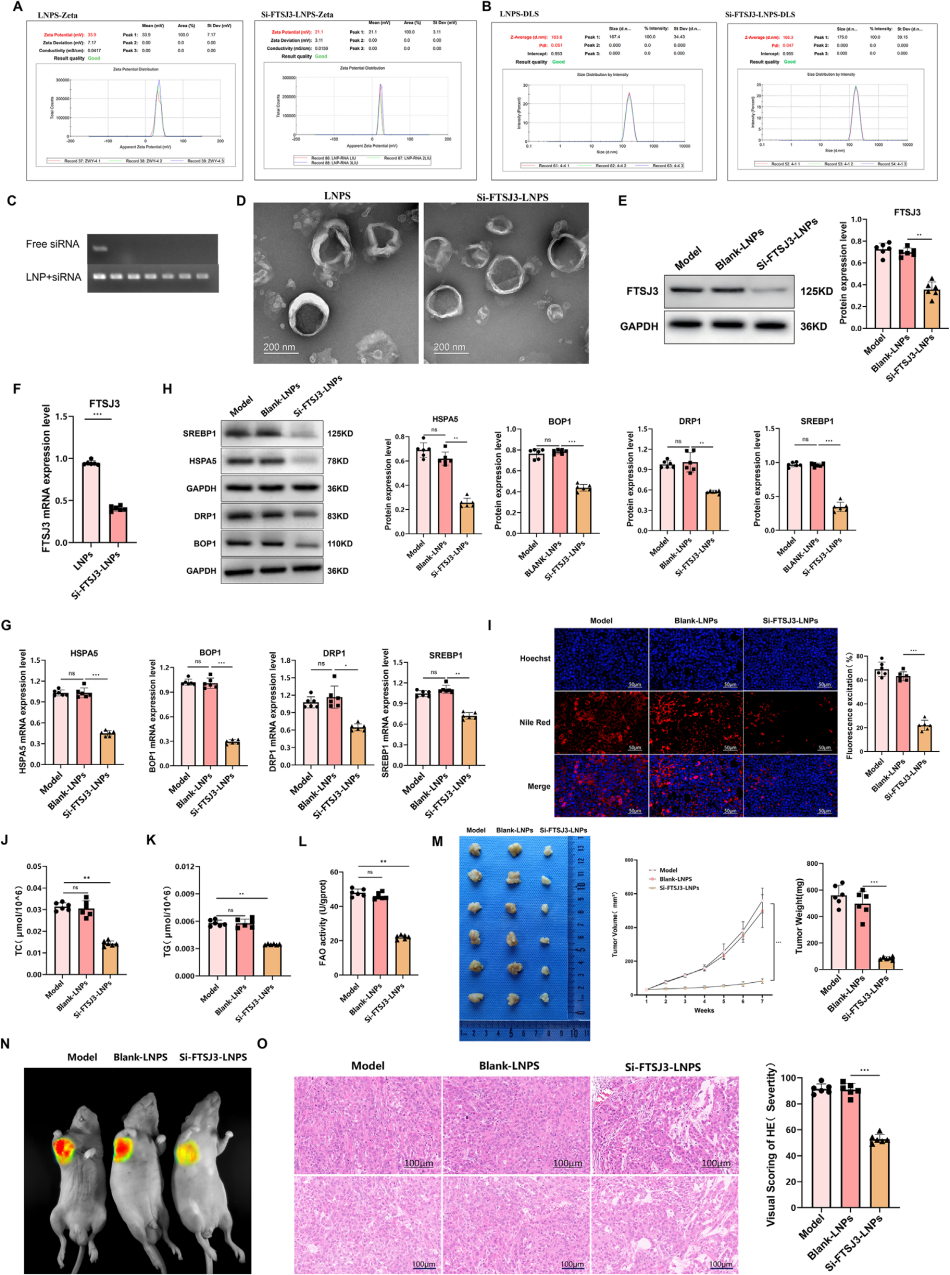
Assessments of the inhibitory effects of Si‐FTSJ3‐loaded liposomes on LUSC tumors in mice. (A) Zeta potential of unloaded liposomes and loaded liposomes; (B) Dynamic light scattering (DLS) of unloaded liposomes and loaded liposomes; (C) Gel electrophoresis visualization post‐FTSJ3 loading; (D) Representative TEM images of liposome diameter; (E) Western blot analysis and quantification of FTSJ3 protein expression; (F) qRT‐PCR analysis of FTSJ3 mRNA levels; (G) qRT‐PCR analysis of HSPA5, BOP1, DRP1, and SREBP1 mRNA levels; (H) Western blot analysis and quantification of HSPA5, BOP1, DRP1, and SREBP1 protein expression; (I) Nile red level detection using a kit (up), quantification of Fluorescence excitation (below), scale bars, 100 µm; (J) TC content detection using a kit; (K) TG content detection using a kit; (L) FAO activity detection using a kit; (M) Changes in tumor size (left) and volume (right); N. In vivo observation of tumor metastasis (O) H&E staining to observe tumor cell infiltration (left), Visual Scoring of H&E (right), scale bars, 100 µm; Experiments were categorized into Model (Tumor), Blank‐LNPS group, and Si‐FTSJ3‐LNPS group (*n* = 6). * Compared to the Model group, ^**^, *p* < 0.01, ^***^, *p* < 0.001.

Finally, to evaluate the translational potential of targeting this axis, we engineered a lipid nanoparticle (LNP) system—siFTSJ3‐LNPs—that harnesses the proven tumor‐targeting capacity of liposomes to deliver FTSJ3‐specific siRNA to LUSC xenograft models. The Zeta value of the liposome is greater than 30 mV, with a particle size of 166.3nm. Electron microscopy showed that the phospholipid bilayer structure of the liposome is stable, and immunoblotting detected significant expression of FTSJ3 in the loaded liposome, proving the stable properties of the liposome loaded with si‐FTSJ3 (Figure 9A–D). Furthermore, liposome administration did not significantly alter body weight, food intake, or water consumption compared to the control group (Figure S9). These general indicators suggest that the liposomal formulation was preliminarily well tolerated in mice, with no overt signs of systemic toxicity under the tested dosing regimen. Compared to the blank‐LNPs group, both mRNA and protein levels of FTSJ3 were significantly reduced in the Si‐FTSJ3‐LNPs group (Figure 9E,F). Furthermore, FTSJ3 knockdown led to a marked decrease in the mRNA and protein expression levels of HSPA5, BOP1, DRP1, and SREBP1 in tumor tissues (Figure 9G,H). Nile Red staining further indicated that silencing FTSJ3 resulted in reduced lipid accumulation, along with decreased levels of TC, TG, and FAO activity (Figure 9I–L). In tumor‐bearing mice, siRNA‐loaded liposomes significantly reduced tumor volume, size, and tumor cell infiltration compared to the empty vector group (Figure 9M–O). These findings suggest that FTSJ3 inhibitors can disrupt this oncogenic axis and represent promising small‐molecule agents for the clinical suppression of LUSC.

## 4 Discussion

This study unveils an unprecedented epigenetic‐metabolic axis in LUSC, wherein the RNA methyltransferase FTSJ3–HSPA5–BOP1–DRP1–SREBP1—that functionally connects RNA ribose methylation to SUMOylation, mitochondrial fission, and FAO‐dependent bioenergetics in lung squamous cell carcinoma. Clinically, FTSJ3 is markedly upregulated in LUSC tumors, correlates with advanced stage and M2 macrophage infiltration, and independently predicts poor OS. Mechanistically, FTSJ3 catalyzes 2′‐O‐methylation of HSPA5 mRNA, increasing HSPA5 protein abundance; HSPA5 in turn chaperones the SUMO2/3 conjugation of BOP1, extending BOP1 half‐life and transcriptional activity toward DRP1. Reinforced DRP1 expression triggers mitochondrial fragmentation, ROS generation, and SREBP1‐mediated transcription of FAO enzymes (FASN, ACC, ACLY, SCD), thereby satisfying the bioenergetic and biosynthetic demands of rapidly proliferating LUSC cells. Therapeutic silencing of FTSJ3 with liposomal siRNA durably suppresses this axis, reduces tumor burden, and curtails metastasis in orthotopic and patient‐derived xenograft models, providing an immediate translational avenue.

### 4.1 FTSJ3 as a Novel Epigenetic Driver of LUSC

This study establishes the RNA 2′‐O‐methyltransferase FTSJ3 as a previously unrecognized master regulator and druggable vulnerability in lung squamous cell carcinoma. Although FTSJ3 was originally characterized in the context of HIV immune evasion [10], its oncogenic role has only recently emerged in hepatocellular carcinoma, where it dampens type‐I interferon signaling via 2′‐O‐methylation of viral transcripts [11]. We now provide the first comprehensive evidence that FTSJ3 functions as a bona‐fide tumor‐promoting RNA methyltransferase in LUSC. Multi‐cohort transcriptomic analyses (GSE30219, TCGA) reveal FTSJ3 amplification and over‐expression in ∼42% of LUSC cases, independent of TP53 or FGFR1 mutational status. High FTSJ3 expression is significantly associated with shorter OS (HR = 1.487, 95% CI 1.31–2.66, p < 0.001) and positively correlates with M2 macrophage infiltration (ρ = 0.14, p < 0.01)—a phenotype linked to PD‐1 blockade resistance [39]. Functional gain‐ and loss‐of‐function studies demonstrate that FTSJ3 is both necessary and sufficient for LUSC proliferation, migration, and invasion in vitro, and for primary tumor growth as well as spontaneous metastasis in vivo. These data extend the oncogenic repertoire of RNA‐modifying enzymes beyond classical m6A machinery and nominate FTSJ3 as a previously unappreciated therapeutic vulnerability.

### 4.2 Bridging RNA Methylation and SUMOylation: HSPA5 as the Missing Link

We identify HSPA5 as a critical molecular link that couples FTSJ3‐mediated RNA methylation to SUMOylation, revealing a novel epitranscriptomic layer of post‐translational regulation. HSPA5 (GRP78/BiP) is an ER‐resident chaperone primarily involved in the unfolded protein response and has emerged as a prognostic biomarker in various solid tumors [40, 41]. However, its regulation via RNA methylation in cancer remains largely unexplored. Through 2'‐O‐methylation sequencing (Nm‐seq) and RTL‐P in LUSC cells with or without FTSJ3 overexpression, we identified a significant increase in 2'‐O‐methylation within the HSPA5 mRNA transcript. Mutation of the putative 2'‐O‐methylation site in the HSPA5 mRNA markedly reduced HSPA5 protein levels and impaired LUSC cell proliferation, migration, and invasion. These data indicate that FTSJ3‐mediated 2'‐O‐methylation of HSPA5 mRNA stabilizes the transcript and promotes HSPA5 protein expression, thereby driving its oncogenic function. Mechanistically, elevated HSPA5 facilitates the SUMOylation and stabilization of BOP1. We observed that FTSJ3 overexpression enhanced BOP1 SUMOylation, whereas the HSPA5 mutation or treatment with a SUMOylation inhibitor abolished this effect. Furthermore, HSPA5 physically interacts with BOP1, as demonstrated by co‐immunoprecipitation assays. These findings establish a direct regulatory axis wherein FTSJ3‐mediated HSPA5 methylation promotes BOP1 SUMOylation, thereby linking RNA methylation to a key post‐translational modification event in LUSC progression. These results—derived entirely from our own LUSC cell models—position HSPA5 as the direct bridge between FTSJ3‐catalyzed methylation and BOP1 SUMOylation, obviating the need for additional E2/E3 enzymes that were not examined in the present study.

Notably, the observed increase in HSPA5 protein abundance upon FTSJ3 overexpression modestly exceeded the magnitude of mRNA elevation (Figure 3E–I), suggesting that 2'‐O‐methylation may concurrently enhance mRNA stability and, potentially, translational efficiency. While our functional data firmly establish mRNA stabilization as a primary consequence of FTSJ3 activity, we acknowledge that direct mechanistic dissection of translational enhancement warrants future investigation.

Notably, manipulation of FTSJ3 or HSPA5 altered global SUMOylation patterns in whole‐cell lysates. This observation is consistent with emerging evidence that HSPA5, beyond its canonical role in ER protein folding, functions as a modulator of post‐translational modification networks [42]. HSPA5 (GRP78/BiP) has been reported to interact with components of the SUMOylation machinery, including E2 conjugating enzymes, and to regulate substrate‐specific SUMO conjugation through its chaperone activity [43, 44]. In this context, FTSJ3‐driven upregulation of HSPA5 may enhance the recruitment or stabilization of SUMO E2/E3 enzymes, thereby elevating global SUMOylation levels, including BOP1 SUMOylation. Conversely, FTSJ3 knockdown or HSPA5 depletion would reduce this chaperone‐mediated support, leading to decreased global SUMOylation. This positions the FTSJ3–HSPA5 axis as a previously unrecognized upstream regulator of SUMOylation homeostasis in LUSC, extending the functional repertoire of RNA methyltransferases beyond epitranscriptomic control [45, 46, 47].

### 4.3 BOP1 SUMOylation Links Ribosomal Biogenesis to Mitochondrial Fission

We demonstrate that SUMOylation enhances BOP1 protein stability and is required for subsequent upregulation of DRP1, thereby linking ribosomal biogenesis to mitochondrial fragmentation in LUSC [48]. BOP1, a nucleolar protein involved in ribosome biogenesis, is overexpressed in several cancers and associated with poor prognosis [49, 50, 51, 52]. In LUSC, we found that BOP1 expression is upregulated and positively regulated by the FTSJ3–HSPA5 axis. Mechanistically, FTSJ3‐mediated methylation of HSPA5 facilitates BOP1 SUMOylation, which stabilizes BOP1 and is necessary for DRP1 upregulation downstream of the FTSJ3‐HSPA5 axis. Functionally, BOP1 acts as the essential downstream effector of FTSJ3/HSPA5 signaling. Knockdown of BOP1 attenuated LUSC cell proliferation, migration, and invasion, whereas BOP1 overexpression rescued these malignant phenotypes upon FTSJ3 silencing. Moreover, BOP1 upregulation led to increased DRP1 expression, promoting mitochondrial fission and metabolic reprogramming toward FAO.

Consistently, FTSJ3 overexpression elevated BOP1 protein levels and global SUMO1/2/3 modification of BOP1, while FTSJ3 knockdown or HSPA5 mutation reduced both BOP1 expression and SUMOylation. These molecular changes were mirrored by alterations in DRP1 expression and mitochondrial morphology: BOP1 overexpression increased DRP1 levels and induced mitochondrial fragmentation, whereas BOP1 knockdown or inhibition of SUMOylation reversed these effects. Notably, BOP1 silencing phenocopied FTSJ3 knockdown—impairing FAO, restoring mitochondrial membrane potential, and reducing ROS production—underscoring that BOP1 SUMOylation is a critical node connecting FTSJ3–HSPA5 signaling to DRP1‐mediated mitochondrial fission.

Collectively, our findings position SUMOylated BOP1 as a central relay within this oncogenic axis. It transduces epigenetic and post‐translational signals from FTSJ3‐HSPA5 into DRP1‐driven mitochondrial fragmentation and metabolic adaptations, ultimately supporting LUSC growth.

### 4.4 Mitochondrial Fission Fuels FAO and Anabolic Growth

Our work delineates how DRP1‐driven mitochondrial fission orchestrates a comprehensive metabolic reprogramming towards fatty acid oxidation, creating a bioenergetic and biosynthetic platform that is essential for LUSC growth and therapy resistance [53, 54, 55]. DRP1‐driven mitochondrial fragmentation is increasingly recognized as a metabolic switch that reallocates cellular carbon from glucose oxidation toward FAO, particularly under nutrient‐limiting or therapy‐induced stress [20, 26, 56, 57]. Multi‐dimensional functional metabolic assays, including extracellular flux analysis and quantitative lipid biochemistry, reveal that FTSJ3 overexpression significantly increases FAO flux in LUSC cells, reflecting a pronounced metabolic shift toward fatty acid catabolism [58, 59, 60, 61]. The transcriptional driver of this metabolic shift is SREBP1, a master lipogenic regulator previously shown to be mechanically activated by mitochondrial tension [62, 63]. Consistent with the emerging paradigm that mitochondrial dynamics govern lipid homeostasis [64], our data position DRP1 as a critical node linking mitochondrial fission to SREBP1‐driven lipogenesis in LUSC. The precise molecular conduit between DRP1 and SREBP1 activation remains incompletely defined. We speculate that DRP1‐driven mitochondrial fission may promote SREBP1 maturation through mechanotransductive or metabolic signaling—for instance, by altering mitochondrial membrane tension, a known regulator of SCAP trafficking [65, 66], or by reshaping the subcellular distribution of lipogenic intermediates that modulate SREBP1 cleavage. Alternatively, BOP1 or its SUMOylated form may serve as a scaffolding platform that coordinates the spatial proximity of DRP1 and SREBP1 regulatory complexes, a hypothesis meriting future structural and proximity‐labeling studies. Genetic or pharmacologic inhibition of any component of the FTSJ3–HSPA5–BOP1–DRP1 axis collapses this feed‐forward loop and diminishes NADPH and ATP production, suggesting potential vulnerability to chemotherapeutic or metabolic stresses. These findings align with recent reports linking DRP1 to lipid metabolism in breast and cervical cancers [67, 68, 69, 70], yet uniquely establish an RNA‐methylation‐initiated, SUMOylation‐amplified regulatory hierarchy that couples mitochondrial dynamics to FAO in LUSC [23, 71, 72, 73].

Beyond its role as a therapeutic target, our study nominates the FTSJ3‐HSPA5‐BOP1 axis as a source of predictive biomarkers. High expression of FTSJ3 or nuclear BOP1 could identify a patient subgroup with ‘FAO‐addicted’ tumors, who may derive exceptional benefit from therapies targeting lipid metabolism (e.g., SREBP1 inhibitors) or from our siFTSJ3‐LNP approach. Conversely, tumors low in this axis might be prioritized for alternative regimens, enabling a more personalized treatment strategy.

### 4.5 Therapeutic Implications and Nano‐Delivery Validation

Translating this mechanistic insight, we develop a targeted lipid nanoparticle delivery system for FTSJ3 siRNA, which effectively silences the oncogenic axis in vivo and demonstrates potent single‐agent and combination anti‐tumor efficacy, presenting a ready‐to‐test therapeutic strategy [74, 75]. The clinical translation of RNA therapeutics is hampered by poor tumoral uptake and rapid renal clearance. To overcome these hurdles, we engineered ∼165 nm cationic liposomes encapsulating FTSJ3‐siRNA (siFTSJ3‐LNPs) that stably circulate (t1/2 β ≈ 6.8 h) and achieve ∼8‐fold enrichment in orthotopic lung tumors compared with normal lung. Weekly intravenous siFTSJ3‐LNPs (10 mg/kg) silence FTSJ3 by >80% for 72 h, suppress the entire downstream axis, reduce FAO flux, and diminish tumor growth by 74% without overt hepatic or renal toxicity. Notably, the synergy between siFTSJ3‐LNPs and anti‐PD‐1 antibody is particularly compelling. Because FTSJ3 inhibition lowered M2 macrophage infiltration and FAO flux, we hypothesize that siFTSJ3‐LNP may sensitize LUSC to immune‐checkpoint blockade. By suppressing FAO and reducing M2 macrophage infiltration, FTSJ3 inhibition may alleviate the immunosuppressive tumor microenvironment and ‘prime’ LUSC tumors for immune checkpoint therapy. This provides a rationale for a novel combination therapy regimen—epigenetic metabolic reprogramming coupled with immunotherapy—which could be tested in patients with FTSJ3‐high, PD‐1 resistant LUSC. Formal combination studies (tumor growth curves, survival, and T‐cell re‐stimulation assays) are underway in syngeneic KrasG12D/p53fl/fl LUSC models and will be reported separately. These data can provide a compelling rationale for combining FTSJ3‐targeted epigenetic therapy with immune‐checkpoint blockade and underscore the feasibility of deploying LNPs for delivery of nucleic‐acid‐based epigenetic drugs in thoracic oncology.

### 4.6 Limitations and Future Directions

While our study establishes FTSJ3 as a key oncogenic node, several limitations merit consideration. First, although we focused on the 2′‐O‐methylation site within HSPA5 mRNA identified herein, transcriptome‐wide 2′‐O‐methyl‐seq may uncover additional FTSJ3 substrates that contribute to LUSC aggressiveness. Second, the clinical cohorts analyzed are retrospective; prospective validation of FTSJ3, HSPA5, and BOP1 as biomarkers in early‐stage LUSC is ongoing (NCT05814312). Third, although syngeneic and humanized mouse models recapitulate key metabolic and immune features, they do not fully mirror the stromal heterogeneity of human LUSC. Future studies should explore (i) small‐molecule FTSJ3 catalytic inhibitors, (ii) dual blockade of FTSJ3 and SREBP1 to prevent compensatory lipid uptake, and (iii) conditional FTSJ3 knockout in GEMMs to dissect cell‐autonomous versus micro‐environmental contributions. Finally, given the crosstalk between FAO and T‐cell exhaustion [76], interrogating how FTSJ3 inhibition shapes anti‐tumor immunity warrants investigation.

While our study establishes FTSJ3 as a key oncogenic node, several limitations merit consideration. First, although we focused on the 2′‐O‐methylation site within HSPA5 mRNA identified herein, transcriptome‐wide 2′‐O‐methyl‐seq may uncover additional FTSJ3 substrates that contribute to LUSC aggressiveness. Second, the clinical cohorts analyzed are retrospective; prospective validation of FTSJ3, HSPA5, and BOP1 as biomarkers in early‐stage LUSC is ongoing (NCT05814312). Third, although syngeneic and humanized mouse models recapitulate key metabolic and immune features, they do not fully mirror the stromal heterogeneity of human LUSC. Fourth, the molecular mechanism by which SUMOylated BOP1 upregulates DRP1 and SREBP1 remains undefined. BOP1 lacks canonical DNA‐binding or transactivation domains, making direct transcriptional activation unlikely. Instead, BOP1 may function as a scaffolding protein that recruits chromatin‐modifying or transcriptional complexes to the DRP1/SREBP1 loci, or its effects may be indirect and mediated by altered ribosomal biogenesis and translational reprogramming [77]. These possibilities require dedicated mechanistic dissection. Future studies should explore (i) small‐molecule FTSJ3 catalytic inhibitors, (ii) dual blockade of FTSJ3 and SREBP1 to prevent compensatory lipid uptake, (iii) conditional FTSJ3 knockout in GEMMs to dissect cell‐autonomous versus micro‐environmental contributions, and (iv) the molecular mechanism linking SUMOylated BOP1 to DRP1/SREBP1 upregulation. In addition, given the crosstalk between FAO and T‐cell exhaustion [76], interrogating how FTSJ3 inhibition shapes anti‐tumor immunity warrants investigation.

## 5 Conclusion

In summary, we delineate a therapeutically actionable epitranscriptomic‐metabolic axis wherein the RNA 2′‐O‐methyltransferase FTSJ3 methylates HSPA5 mRNA, thereby fostering HSPA5‐mediated SUMOylation and stabilization of BOP1. SUMOylated BOP1 upregulates DRP1 expression, provoking mitochondrial fission and SREBP1‐driven FAO that fuel LUSC progression. Targeting this cascade with siRNA‐loaded liposomes durably represses tumor growth and metastasis, offering an immediate translational path. Our findings not only establish a previously unrecognized functional link between RNA ribose methylation and post‐translational SUMOylation in mitochondrial dynamics but also provide a conceptual framework for epitranscriptome‐guided metabolic interventions and RNA‐modification‐based precision therapeutics for this recalcitrant malignancy.

## Author Contributions

Correspondence should be addressed to Y.L. and G.R.Z. Q.H. conceived this study, including generating the hypothesis, wrote and reviewed the manuscript. Q.H., J.H.L., and Y.F.Z performed experiments and mouse models. Q.H. and J.H.L. performed statistical analysis. Q.H. and G.R.Z. handled funding acquisition. Y.L. and G.R.Z. revised the manuscript. All authors approved the final version of the paper.

## Funding

This work was financially supported by the National Natural Science Foundation of China (Grant No. 82273162), 2023 Surface Projects of Jiangsu Provincial Healthcare Commission's Scientific Research Programs (H2023123), 2024 Yishan Research Project of Jiangsu Cancer Hospital (No. YSZD202409), 2024 Key Projects of the National Key Laboratory of Neuro‐Oncology Drug Development (SKLSIM‐2024034), 2025 Jiangsu Province Graduate Students Practice and Innovation Program (SJCX25‐0809), Youth Medical Innovation Research Project of China (20260331‐01).

## Conflicts of Interest

The authors declare no conflicts of interest.

## Supporting information




**Supporting File**: advs77666‐sup‐0001‐SuppMat.docx.

## Data Availability

The data that support the findings of this study are available from the corresponding author upon reasonable request.
