# Supplementary material for: FTSJ3‐Mediated 2′‐O‐Methylation of HSPA5 mRNA Stabilizes BOP1 via SUMOylation to Drive DRP1‐Dependent Mitochondrial Fission and Fatty Acid Oxidation in Lung Squamous Cell Carcinoma
Source: Adv Sci (Weinh). 2026 Sep 8:e77666. Online ahead of print. doi: 10.1002/advs.77666 (PMC13554496; doi:10.1002/advs.77666)
Supplement: Supplementary file 1 [file ADVS-9999-e77666-s001.docx]

**Supplemental Figures and Figure Legends**

***Figure S1 The mRNA levels of FTSJ3, DNMT3B, NCBP2, and IGF2BP2 in LUSC tumor and adjacent non-tumor tissues.***

qRT-PCR analysis of FTSJ3, DNMT3B, NCBP2, and IGF2BP mRNA levels in LUSC tumor and adjacent non-tumor tissues. Experiments were divided into Control group (adjacent non-tumor tissues) and Tumor group. *, compared to Control group, **, p < 0.01, ***, p < 0.001.

***Figure S2 The expression of HSPA5 in tumor tissue and adjacent normal tissue.***

Box plot of HSPA5 expression in tumor tissue and adjacent normal tissue.

***Figure S3 The expression of BOP1 in tumor tissue and adjacent normal tissue.***

Box plot of BOP1 expression in tumor tissue and adjacent normal tissue.

***Figure S4 Overexpression of FTSJ3 enhances the interaction between BOP1 and HSPA5.***

A. The PPI network was constructed based on transcriptomic data from LUSC. B. The levels of BOP1 on HSPA5 were assessed by Co-IP assays in H520 cells treated with OE-FTSJ3 or pcDNA3.1.

***Figure S5 The SUMOylation level of BOP1***

A. The SUMOylation level of BOP1 were assessed by Co-IP assays in H520 cells treated with OE-FTSJ3 or pcDNA3.1. B. The SUMOylation level of BOP1 were assessed by Co-IP assays in H520 cells treated with pcDNA3.1, OE-FTSJ3, si-NC or siHSPA5.

***Figure S6 The mRNA levels of BOP1 in SKMES cells.***

SKEMS cells were treated with Si-BOP1. qRT-PCR analysis of BOP1 mRNA levels in SKEMS cells. *, compared to Control group, **, p < 0.01.

***Figure S7 BOP1 promotes FAO metabolism via SREBP1 in H520 Cells.***

H520 cells were treated with Si-SREBP1, Si-NC, OE-SREBP1 or pcDNA3.1 after treated with OE-BOP1. A. Western blot analysis and quantification of DRP1 and SREBP1 protein expression. B. qRT-PCR analysis of DRP1 and SREBP1 mRNA levels. C. Nile red level detection using a kit (left), quantification of Fluorescence excitation (right), scale bars, 100 μm; D. TC content detection using a kit; E. TG content detection using a kit; F. FAO activity detection using a kit; G-J. qRT-PCR analysis of FASN, ACC, ACLY, and SCD mRNA levels. K. Western blot analysis and quantification of FASN, ACC, ACLY, and SCD protein expression. Experiments were divided into BOP1 overexpression combined with knockdown control group (OE-BOP1+Si-NC), BOP1 overexpression combined with overexpression plasmid control (OE-BOP1+pcDNA3.1), BOP1 overexpression combined with SREBP1 knockdown group (OE-BOP1+Si-SREBP1), and BOP1 overexpression combined with SREBP1 overexpression group (OE-BOP1+OE-SREBP1). * Compared to the OE-BOP1+Si-NC or OE-BOP1+pcDNA3.1 group, *, p < 0.05, **, p < 0.01, ***, p < 0.001.

***Figure S8 BOP1 promotes FAO metabolism via SREBP1 in LUSC Cells.***

H520 and SKMES cells were treated with OE-SREBP1 or pcDNA3.1 after treated with si-BOP1. A and B. Western blot analysis and quantification of DRP1 and SREBP1 protein expression. C and D. Nile red level detection using a kit (left), quantification of Fluorescence excitation (right), scale bars, 100 μm; E and H. TC content detection using a kit; F and I. TG content detection using a kit; G and J. FAO activity detection using a kit; K and L. Western blot analysis and quantification of FASN, ACC, ACLY, and SCD protein expression. Experiments were divided into knockdown control group (Si-NC)

, BOP1 knockdown group (si-BOP1), knockdown BOP1 combined with SREBP1 overexpression group (si-BOP1+OE-SREBP1), and BOP1 knockdown combined with SREBP1 knockdown group (si-BOP1+si-SREBP1). * Compared to the si-NC or si-BOP1+ si-SREBP1 group, *, p < 0.05, **, p < 0.01, ***, p < 0.001.

***Figure S9 Evaluation of the toxicity of Si-FTSJ3-loaded liposomes in mice.***

A. The body weight of mice. B. The food intake of mice. C. The water intake of mice. Experiments were categorized into Model (Tumor), Blank-LNPs group, 5 mg/kg LNPs group, 10 mg/kg LNPs group and 20 mg/kg LNPs group (n=6). * Compared to the Blank-LNPs group, ns, no significance.

***Table S1 The PCR primers***

| Gene | Uptream primer sequence | Downstream primer sequence |
| --- | --- | --- |
|  |  |  |
| FTSJ3 | GTGCACCATCATTCCGCCCTGGAGG | GTGCGTTGTTCTCACCTGAATCACC |
| BOP1 | GAT AGC AAG CTG GTG TGG TTT | CGC CAC AAG ATG CAC GTA CCT |
| HSPA5 | ACC AGG AAT GAC TTC TAG TTTGA | GGG TTT GCC GGG GTA CAA TA |
| DRP1 | ACCTACCCAGTGCCAGCCAGT | ATCAAACACGGTGAAGTACAGCCTCAGGA |
| ACLY | AGGTGTGAGCCACCAAGACT | CAGCCCAGAATGCAGGGAAG |
| FASN | GGAGGTGGTGATAGCCGGTAT | TGGGTAATCCATAGAGCCCAG |
| SREBP1 | TGCCCAGCACGTTGCGCCCA | TCCATGGTCACTTTCCGGGA |
| GRPDH | GATTCCACCCATGGCAAATTC | CTGGAAGATGGTGATGGGATT |

***Table S2 The 2′-O-methylation of HSPA5 primer***

| Gene | Primers sequence | |
| --- | --- | --- |
| HSPA5 | F: AGAAGGTTACCCATGCAGT | R: AGGCCAGCAATAGTTCCA |
